# Long-term assessment of floodplain reconnection as a stream restoration approach for managing nitrogen in ground and surface waters

**DOI:** 10.1007/s11252-021-01199-z

**Published:** 2022-01-07

**Authors:** Paul M. Mayer, Michael J. Pennino, Tammy A. Newcomer-Johnson, Sujay S. Kaushal

**Affiliations:** 1 US Environmental Protection Agency, Office of Research and Development, Center for Public Health and Environmental Assessment, Pacific Ecological Systems Division, Corvallis, OR 97333, USA; 2 US Environmental Protection Agency, Office of Research and Development, Center for Public Health and Environmental Assessment, Health and Environmental Effects Assessment Division, Integrated Environmental Assessment Branch, Washington DC 20460, USA; 3 US Environmental Protection Agency, Center for Environmental Measurement and Modeling, Watershed and Ecosystem Characterization Division, 26 W. Martin Luther King Drive, Cincinnati, OH 45268, USA; 4 Department of Geology & Earth System Science Interdisciplinary Center, University of Maryland, College Park, MD 20740, USA

**Keywords:** Chloride, Denitrification, Floodplain reconnection, Geomorphology, Hydrology, Groundwater, Minebank run, Nitrate, Resilience, Restoration, Surface water, Urban stream

## Abstract

Stream restoration is a popular approach for managing nitrogen (N) in degraded, flashy urban streams. Here, we investigated the long-term effects of stream restoration involving floodplain reconnection on riparian and in-stream N transport and transformation in an urban stream in the Chesapeake Bay watershed. We examined relationships between hydrology, chemistry, and biology using a Before/After-Control/Impact (BACI) study design to determine how hydrologic flashiness, nitrate (NO_3_^−^) concentrations (mg/L), and N flux, both NO_3_^−^ and total N (kg/yr), changed after the restoration and floodplain hydrologic reconnection to its stream channel. We examined two independent surface water and groundwater data sets (EPA and USGS) collected from 2002–2012 at our study sites in the Minebank Run watershed. Restoration was completed during 2004 and 2005. Afterward, the monthly hydrologic flashiness index, based on mean monthly discharge, decreased over time from 2002 and 2008. However, from 2008–2012 hydrologic flashiness returned to pre-restoration levels. Based on the EPA data set, NO_3_^−^ concentration in groundwater and surface water was significantly less after restoration while the control site showed no change. DOC and NO_3_^−^ were negatively related before and after restoration suggesting C limitation of N transformations. Long-term trends in surface water NO_3_^−^ concentrations based on USGS surface water data showed downward trends after restoration at both the restored and control sites, whereas specific conductance showed no trend. Comparisons of NO_3_^−^ concentrations with Cl^−^ concentrations and specific conductance in both ground and surface waters suggested that NO_3_^−^ reduction after restoration was not due to dilution or load reductions from the watershed. Modeled NO_3_^−^ flux decreased post restoration over time but the rate of decrease was reduced likely due to failure of restoration features that facilitated N transformations. Groundwater NO_3_^−^ concentrations varied among stream features suggesting that some engineered features may be functionally better at creating optimal conditions for N retention. However, some engineered features eroded and failed post restoration thereby reducing efficacy of the stream restoration to reduce flashiness and NO_3_^−^ flux. N management via stream restoration will be most effective where flashiness can be reduced and DOC made available for denitrifiers. Stream restoration may be an important component of holistic watershed management including stormwater management and nutrient source control if stream restoration and floodplain reconnection can be done in a manner to resist the erosive effects of large storm events that can degrade streams to pre-restoration conditions. Long-term evolution of water quality functions in response to degradation of restored stream channels and floodplains from urban stressors and storms over time warrants further study, however.

## Introduction

Urban streams receive excess nitrogen (N) from multiple sources in the watershed and transport N downstream because supply from the watershed is greater than demand in the stream (Grimm et al. 2005; Kaushal et al. 2014a, b). Channel degradation from flashy runoff, incision, and floodplain disconnection impairs N transformation and uptake and exacerbates downstream N transport (Paul and Meyer 2001). Consequently, stream restoration designed to repair and reconnect stream channels, is an increasingly popular approach for managing N in urban streams. Such restoration attempts to improve hydrologic condition favorable for N transformation and denitrification, by reducing flashiness, increasing residence times, and adding organic carbon for denitrifiers (Kaushal et al. 2008; Gift et al. 2010; Mayer et al. 2010b; Duan et al. 2019). Our recent synthesis suggested that there is potential for reducing N through stream restoration (Newcomer Johnson et al. 2016), though it is not clear which methods are most effective. Identifying effective watershed restoration and stormwater BMPs is critical for establishing protocols to meet nutrient management goals in watersheds like the Chesapeake Bay, USA where this study was conducted. (Urban Stormwater Work Group 2020). While some restoration approaches have shown short-term benefits (Bukaveckas 2007; Kaushal et al. 2008; Filoso and Palmer 2011; Filoso et al. 2015), most projects receive surprisingly little or no post-restoration assessment (Bernhardt et al. 2005; Hassett et al. 2005). Most stream restoration studies are short-term evaluations employing space for time substitutions examining restored and unrestored, reference sites simultaneously (but see Bukaveckas 2007). Even fewer studies have examined groundwater-surface water interactions in urban or restored streams (Striz and Mayer 2008; Mayer et al. 2010b). Our research was intended to fill a gap in long-term studies of restoration, improve our understanding of N behavior in groundwater and surface water of restored streams, and elucidate possible BMPs for N management in urban ecosystems.

We investigated the long-term effects of geomorphic stream restoration on riparian and in-stream N transport and transformation at Minebank Run. Initially, Minebank Run was a geomorphically degraded urban stream near Baltimore, Maryland, USA, in the Chesapeake Bay watershed. However, it was later restored by hydrologically reconnecting the stream channel with the floodplain to dissipate erosive force (Kaushal et al. 2008). Over almost a decade, we examined relationships between hydrology, chemistry, and biology before and after channel and floodplain restoration. Before restoration, Minebank Run exhibited “urban stream syndrome” characteristics (Walsh et al. 2005), which included highly eroded stream banks and flashy hydrology stemming from increased runoff from impervious surfaces (Doheny et al. 2006). Channel incision and meandering had exposed buried stormwater and sewer infrastructure prompting Baltimore County Department of Environmental Protection and Sustainability (BCDEPS) to restore Minebank Run. Minebank Run was restored in two phases: (phase 1) an upstream section in 1999 and (phase 2) a downstream section in 2004 and 2005. We hypothesized that stream restoration designed to reconnect the stream to its floodplain would reduce surface and groundwater NO_3_^−^ concentration by creating hydrologic conditions that positively affect microbial activity such as denitrification, improve stream bank stability, and expand hyporheic zones (Mayer et al. 2010b).

Naturally flowing stream channels are hydrologically connected to their floodplains. Urban streams often are disconnected from their floodplains because of stream channel incision from flashy stormwater runoff (Paul and Meyer 2001), altered subsurface flow from engineered urban karst (Kaushal and Belt 2012), and stream burial in pipes (Elmore and Kaushal 2008; Beaulieu et al. 2014, 2015; Pennino et al. 2014). However, increasing hydrologic connectivity at the groundwater-surface water interface can foster “hot spots” and “hot moments” of N removal via denitrification when proper redox conditions develop and when inorganic N and organic C are available to denitrifying bacteria in the subsurface sediments (Hedin et al. 1998; Sobczak and Findlay 2002; Sobczak et al. 2002; McClain et al. 2003; Mayer et al. 2010b; Vidon et al. 2010). The current stream restoration protocol in the Chesapeake Bay region focuses on reconnecting floodplains as the most effective way to increase N uptake in urban streams (Urban Stormwater Work Group 2020). To test the efficacy of floodplain reconnection to reduce N in groundwater and surface water of degraded streams, we examined two independent groundwater and surface water datasets collected at our intensively monitored study site. We targeted stream reaches for intensive study where channel geomorphology was reengineered to reconnect hydrology. We use both empirical and modeling approaches to corroborate results and examined our site both before and after restoration. We examined two different post-restoration time periods (2002–2008 and 2002–2012) because there was evidence for failure of restoration features after 2008. Our results are intended to inform future stream restoration efforts designed to manage N in urban ecosystems by analyzing long-term data, which is rarely available for restored sites.

## Methods

### Study design

We investigated how stream restoration influenced NO_3_^−^ flux and concentration in groundwater and surface water at Minebank Run (Baltimore County, Maryland, USA) from November 2001 to October 2008. Studies of biogeochemistry, geomorphology, hydrology, salinity dynamics, and denitrification processes at Minebank Run have been published elsewhere (e.g., Mayer et al. 2003, 2010b, 2013; Groffman et al. 2005; Doheny et al. 2006, 2007, 2012; Kaushal et al. 2008; Gift et al. 2010; Striz and Mayer 2008; Klocker et al. 2009; Newcomer et al. 2012; Harrison et al. 2011, 2012a, b, 2014; Cooper et al. 2014; Pennino et al. 2016; Wood et al. this issue). Here, we sought to place our research within the context of assessing the restoration of ecosystem processes, hydrologic transport, and the identification of factors limiting and influencing those processes and transport (Palmer 2009). Our specific objectives were to examine relationships among DOC, NO_3_^−^, and Cl^−^ in surface water and groundwater pre- and post-restoration by employing a Before/After-Control/Impact (BACI) study design (Underwood 1992; Thompson et al. 2018). Our study used both long-term monitoring of a stream before and after restoration with intensive groundwater and surface water characterization. We used two independent data sets to produce empirical and modeling results, including our own empirical data (EPA) and from the US Geological Survey (USGS) stream gage data to develop corroborative N flux models spanning pre- and post-restoration study periods. We expected that N transport and processing would be controlled by geomorphology, hydrology, and carbon supply.

### Study area

**Fig. 1:**
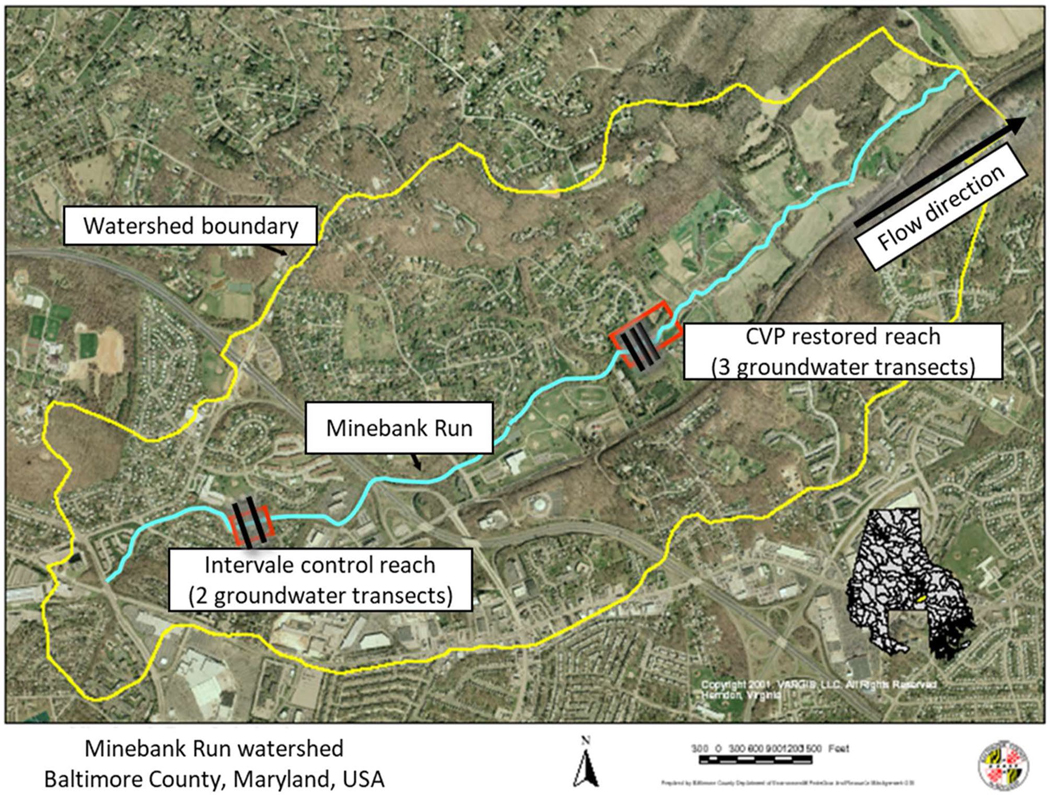
Aerial view of Minebank Run watershed and stream showing locations (red boxes) of study sites. Yellow line delineates the watershed. Blue line represents the stream. Stream flow is to the northeast where Minebank Run confluences with Gunpowder Falls. (Aerial photo courtesy of Baltimore County Department of Environmental Protection and Sustainability)

Minebank Run is a 2 ^nd^ order urban stream located within an 8.47 km^2^ watershed within Baltimore County, Maryland, USA in the eastern section of the Piedmont physiographic province (39^o^24′43″N and 76^o^33′12″W; Fig. 1). Minebank Run flows in a northeasterly direction at approximately a 1% grade for 5.2 km, where it enters Gunpowder Falls, eventually draining into Chesapeake Bay (Doheny et al. 2006). Land use in the Minebank Run watershed is over 80% urban/suburban (Doheny et al. 2006). Between 1960–70 s, rapid urbanization led to severe channel degradation that was addressed by installing concrete flumes in the channel, which, by the 1990s, had been eroded out of place (Sortman 2004). The high proportion of impervious surface in the watershed, including the Interstate-695 Beltway, in a region of significant topographic relief, combined to produce flashy hydrology, eroded banks, incised channel bed, and general geomorphic instability. Hydrographs of storm events at Minebank Run (Doheny et al. 2006) are typical of urban streams in areas of high impervious surface (Paul and Meyer 2001).

Urban development around Minebank Run predates stormwater management regulations implemented in mid-1980’s, and thus, uncontrolled runoff entering the stream was a significant water quality problem. Portions of the channel were encased in concrete, thereby increasing the flashiness of storm flows. Sewer lines and storm drains were eroded and exposed. Riparian buffers were cleared for residential and commercial development.

**Fig. 2:**
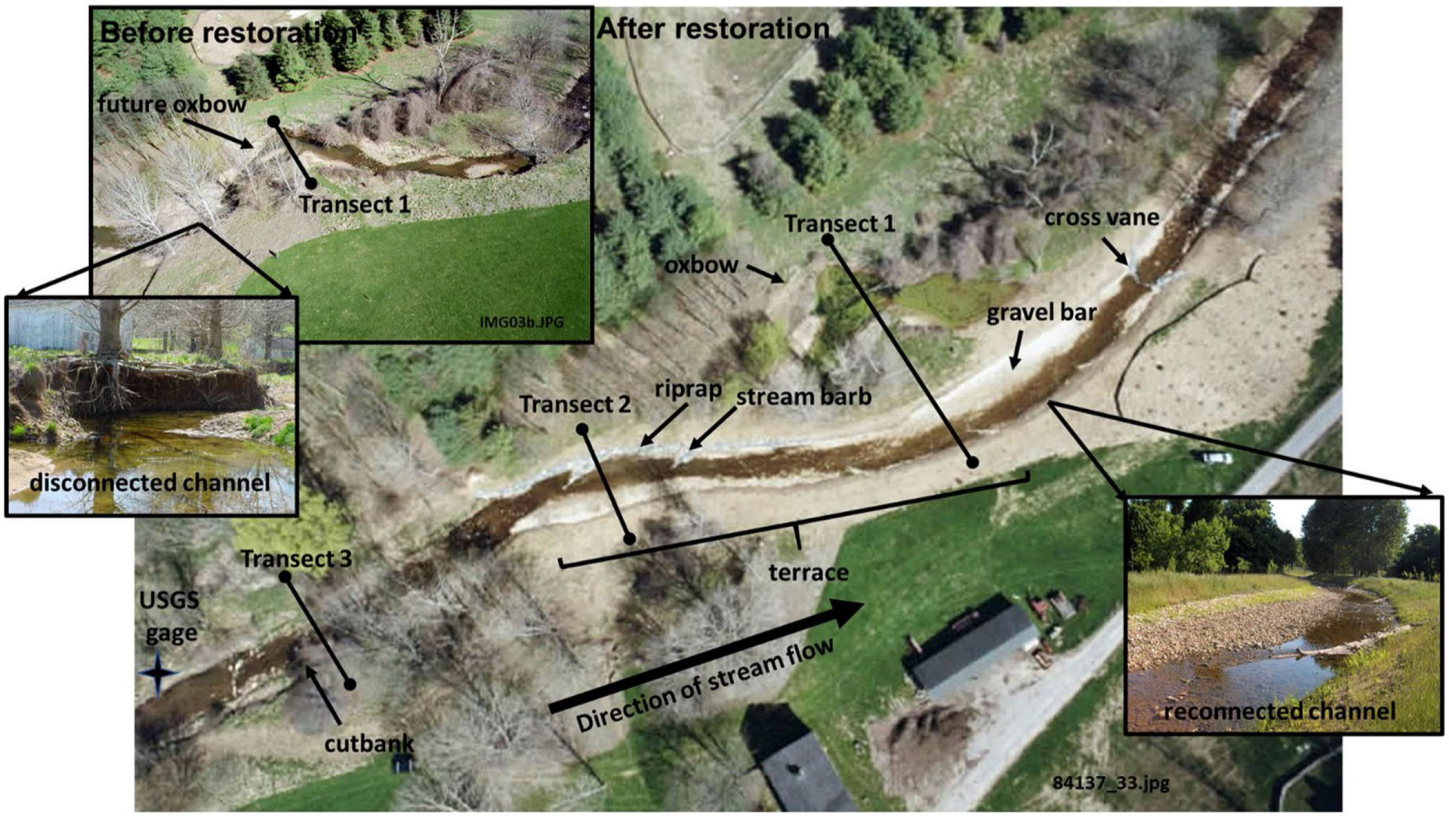
Aerial photos of a portion of the downstream study reach of Minebank Run showing the reconstructed stream before and after the restoration (aerial photos by K. Jewell). Note the bend in the stream channel (future oxbow) that became the oxbow after the restoration

Before restoration, the study reach was characterized by channel incision, revealing bedrock in some places, and causing lateral movement of the stream that impacted property and sewer infrastructure. Pre-restoration channel width ranged from 0.15–15.5 m, and depth ranged from 0.04–0.9 m, yielding channel cross-sectional areas ranging from 0.007 – 13.908 m ^2^ (Doheny et al. 2006). Mean bank height along the study reach was 0.77 ± 0.11 m (Mayer 2010b) with some extremely incised banks up to 1.5 m (Fig. 2). Minebank Run was classified as a B4c channel type (Rosgen 1996), characterized by single-thread channel with entrenchment ratio of 1.4–2.2, width-to-depth ratio > 12, moderate sinuosity > 1.2, water-surface slope of 3–4%, and a median particle diameter in the gravel range of 2 to 64 mm (Doheny et al. 2007).

Phase I of the restoration, which addressed the upstream 2,400 m of stream beginning from the headwaters, began in 1999 and was completed in 2002. Phase II of the restoration, lasting from June 2004 to February 2005, addressed the remaining 3,300 m of the stream to the confluence of the Gunpowder River (USEPA 2006; Doheny et al. 2012). The restored study site is in Cromwell Valley Park (CVP) in approximately the middle of the 3300 m of the Phase II restoration reach and is referred to as restored site (Fig. 1). The control study site is in the Intervale (IV) neighborhood of Towson, MD, USA in approximately the middle of the 2400 m phase I restoration reach (Fig. 1). We refer to this as the control rather than a reference site because it is not pristine (*sensu* Brinson and Rheinhardt 1996), yet it is within the same watershed and exposed to the same runoff and conditions as the restored site. Rather, the control IV site represents a reach that was not manipulated while the downstream site was manipulated by the process of restoration. Our objective was to determine if the downstream reach developed N retention, transport, and/or transformation behavior that was different after the restoration. The control and restored reaches are 2.4 km apart. The restoration included re-engineering approximately 1800 m of mainstem channel and 600 m of tributary channels (Sortman 2004).

Throughout the 5.2 km length of Minebank Run, various stream restoration techniques were used based on the condition of the channel and surrounding land use and infrastructure. For example, the stream channel was redesigned to move the thalweg away from an exposed sewer line to protect against further erosion and channel meandering (Fig. 2). At two points, the channel was redesigned to reduce bank erosion by creating oxbow wetlands (Harrison et al. 2012a, b, 2014), which effectively straightened the channel but allowed for greater overbank flow and stormwater retention. Prior to restoration, about 40–70% of the reach was riffles with runs and pools making up the remainder whereas, after restoration, the proportions of riffles, runs, and pools were more equitable at about 30–40% each (Doheny et al. 2012).

The restoration was intended to reconnect the stream channel with the floodplain by mimicking natural valley and floodplain morphology. For example, the project included root wads and imbricated riprap to reduce bank erosion, cross vanes, step-pools, and pool-riffle features to slow stream flow, as well as a stable meander pattern and cross-section. Natural channel design methods (Rosgen 1996, 2011) were also applied to control flow and erosion by: a) raising the stream bed by filling the channel with gravel and cobble, b) removing concrete liners, c) reconstructing point bars, riffles and meander features, d) creating step-pool structure, e) armoring banks, f) creating oxbow wetlands, and g) re-vegetating the riparian zone (Sortman 2004; Duerksen and Snyder 2005). The restoration corresponded to typologies A, C, and I as described in Newcomer-Johnson et al. (2016) where the stream bottom was raised (A), the floodplain lowered (C), and oxbow wetlands created (I).

**Fig. 3:**
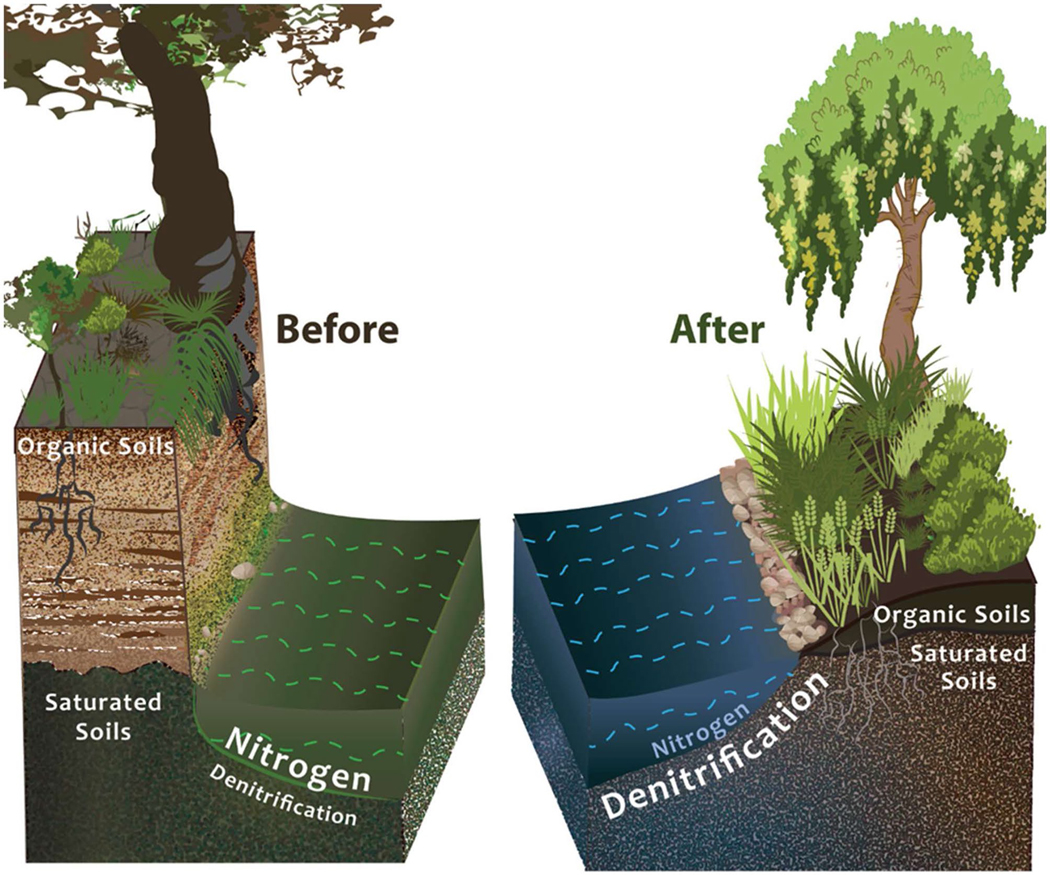
Conceptual figure of stream restoration to improve floodplain reconnection. Eliminating incision and reconnecting the floodplain unites saturated soils with organic matter from plants and roots and allows greater stream channel and groundwater interaction leading to more denitrification and subsequent reductions in stream nitrogen

Although the approach to stream restoration at Minebank Run was primarily intended to address channel erosion and protect sewer infrastructure, we hypothesized that restoration could also affect the hydrology and biogeochemistry of the system (Mayer et al. 2003; Fig. 3). Specifically, we speculated that the physical manipulations designed to accommodate the change in stream discharge rates would also have the potential to change surface and groundwater hydrology (Bukaveckas 2007; Tague et al. 2008). Changes in hydrology could reduce the hydrologic drought (drying of riparian zones due to hydrologic disconnection with the stream and groundwater flowpaths) common in urban streams (Groffman et al. 2003). We speculated that the approach of reshaping the banks and raising the stream bed to eliminate bank incision also might allow carbon-rich riparian soils to become saturated and/or remain wetter, resulting in biogeochemical conditions favorable for nutrient transformations such as denitrification (Kaye et al. 2006). Specifically, we expected flow control structures installed in the stream channel to reduce erosion also may trap organic matter long enough to create enriched anoxic zones conducive for denitrification to occur (Groffman et al. 2005). We also expected re-vegetating the riparian zone could provide litter and organic matter inputs and root biomass to supply carbon to denitrifiers (Gift et al. 2010).

### Precipitation and hydrology

Daily, monthly, and annual precipitation data for the study period (2002–2012) was downloaded from the PRISM Climate Group (https://prism.oregonstate.edu/explorer/; latitude: 39.4200/−76.5788). Stream discharge (m^3^/s) was measured at 5-min intervals at Minebank Run restored CVP with an automated USGS stream gage (USGS ID 0,158,397,967, Minebank Run near Glen Arm, Maryland, USA; http://waterdata.usgs.gov/nwis/uv?0158397967) by the USGS from 2002–2012. To construct regression models of the relationship between discharge and chemistry, the date of the water chemistry sampling was matched with the same date for the mean daily stream discharge provide by the USGS stream gage. While there is no stream gage at the control IV site, discharge measurements and samples collected by USGS are available (USGS ID 0158397925); https://waterdata.usgs.gov/md/nwis/inventory/?site_no=0158397925).

### Seasonal groundwater and surface water chemistry

Groundwater and surface water were collected during 19 sampling events at the restored CVP reach (2001–2008) and 14 corresponding sampling events at the control IV reach (2003–2008). We refer to these samples as EPA data. Sampling dates before 26 May 2004 are considered pre-restoration and sampling dates after 2 March 2005 are post-restoration. At CVP, groundwater and surface water were collected 9 times pre-restoration (28–30 Nov 2001, 5–7 March 2002, 6–9 May 2002, 22–25 July 2002, 15–17 Oct, 2002, 7–9 April 2003, 29–31 July 2003, 15–17 Oct 2003, 10–12 May 2004,) and 10 times post-restoration (29 Nov-1 Dec 2005, 24–27 April 2006, 28–30 Aug 2006, 5–6 Dec 2006, 16–19 April 2007, 26–27 July 2007, 27–29 Nov 2007, 21–24 April 2008, 7–8 July 2008, 27–29 Oct 2008). Groundwater and surface water were collected at control IV beginning April 2003 and continuing through Oct 2008 for the same 14 sampling dates as restored CVP during that period.

Groundwater was collected from the piezometers using low-flow pumping methods (Puls and Barcelona 1996) with a peristaltic pump through a flow cell and multi-meter instrument (Hach Co., Loveland, CO, USA). Surface water was collected via peristaltic pump for consistency with groundwater sampling. Field measurements for all samples included dissolved oxygen (DO; mg/L), pH, temperature (Cº), oxidation reduction potential (ORP; mV), and specific conductance (mS/cm). Samples for C, N, and ion analysis were stored on ice and acidified to pH 2 and/or filtered with 0.45-micron filters, until they could be analyzed in the lab.

Piezometers were installed along transects aligned perpendicular to stream flow in groups of 3 (one group in the channel, and one group each on either bank) at 61, 122, and 183 cm below the surface to capture longitudinal and lateral flow (as described in Striz and Mayer 2008; Kaushal et al. 2008). Transects crossed the stream at geomorphic and restoration features of interest including: cutbanks, gravel bars, terrace, riprap, and oxbows (Fig. 2). A total of 18 piezometers and 2 surface water stations were sampled at our control site at Intervale (IV) and 33 piezometers and 3 surface water stations were sampled at our restored site at Cromwell Valley Park (CVP). At control site IV, piezometers were arranged in 2 transects located 38 m apart across 2 consecutive meander bends. At restored CVP, piezometers were located downstream of USGS stream gage 0,158,397,967 (Fig. 2) and arranged in 3 transects (71 m and 49 m apart). After restoration, some piezometers were replaced at the approximate original pre-restoration locations, where possible, or in comparable locations along the new post-restoration channel. Restoration involved redesigning the channel to fill heavily incised reaches that threatened damage to sewer infrastructure and, in the process, two bends in the channel were cut off to create oxbow wetlands (Fig. 2) that were the focus of previous studies (Harrison et al. 2011, 2012a, b, 2014).

### Bi-weekly surface water chemistry

Independent of the 19 groundwater EPA sampling episodes described above, a second set of surface water samples for NO_3_^−^ and specific conductance was collected by USGS (https://www.usgs.gov/mission-areas/water-resources/science/national-field-manual-collection-water-quality-data-nfm?qt-science_center_objects=0#qt-science_center_objects) approximately every 2 weeks at restored CVP (*N* = 278 sampling events) beginning 5 March 2002 and beginning 3 June 2004 at control IV (*N* = 201 sampling events), and continuing at both sites until 30 June 2008, capturing both pre- and post-restoration periods.

### Laboratory chemical analyses

Chemical analyses followed methodology described in APHA (1998), USEPA (1983), and standard operating procedures (e.g. K-GCRD-SOP-1151–1) performed at the Robert S. Kerr Environmental Research Center, US Environmental Protection Agency (EPA; https://www.epa.gov/research/science-action-roberts-kerr-environmental-research-center-ada-oklahoma) and at USGS (USGS National Water Quality Laboratory: https://www.usgs.gov/labs/nwql). Dissolved organic carbon (DOC) was measured directly on a Tekmar–Dohrmann instrument (Teledyne Technologies Inc., Los Angeles, CA, USA) via the UV-persulfate digestion method. Nitrite ( NO_2_¯) and nitrate ( NO_3_¯) were measured using a Lachat Flow Injection Analyzer (Hach Co., Loveland, CO, USA). Because nitrite was negligible in our samples, we refer to combined nitrite and nitrate as nitrate (NO_3_¯). Cl¯ was measured using capillary electrophoresis with indirect UV detection (Waters Corp., Milford, MA, USA).

### Estimation of N fluxes and hydrologic metrics

Changes in NO_3_^−^ and total nitrogen (TN) flux (kg/yr) were estimated using the R package EGRET (Exploration and Graphics for RivEr Trends) created by the USGS (Hirsch and De Cicco 2015), along with mean daily discharge and concentration data collected every 2 weeks at the Minebank Run stream gage (USGS 0,158,397,967) by the USGS from 2002–2008 and by Pennino et al. (2016) from 2010–2012. The EGRET package applies the Weighted Regressions on Time, Discharge, and Season (WRTDS) smoothing method (Hirsch et al. 2010) for obtaining estimates of annual, monthly and daily flux, and annual flow normalized (FN) flux and concentrations. Flow normalization is used to remove the impact of year-to-year variations in discharge, which helps in assessing changes over time due to changes in the watershed (Medalie et al. 2012). For the FN flux, the mean daily discharge for a particular day is averaged across all years of the data and then multiplied by the concentration on that day instead of just using the concentration and mean daily Q of that particular day (Medalie et al. 2012).

We calculated the flashiness index, a metric to assess the variability in mean daily discharge over a given period of time (Baker et al. 2004; Poff et al. 2006; Sudduth et al. 2011; Violin et al. 2011; Pennino et al. 2016). Greater hydrologic flashiness would indicate more rapid changes in discharge from one day to the next, which is more typical in urban streams in watersheds with high impervious surface compared to less developed or forested watersheds (Konrad et al. 2005; Walsh et al. 2005; Meierdiercks et al. 2010; Smith et al. 2013; Loperfido et al. 2014). Additionally, we applied the same methodology to precipitation data to calculate a “precipitation flashiness index,” based on mean daily rainfall data.

NO_3_^−^ flux and the flashiness index were calculated over two different time periods: 1) using just the USGS concentration dataset from 2002–2008 and 2) including an additional concentration dataset collected from 2010–2012 (Pennino et al. 2016). The NO_3_^−^ and TN concentrations were taken from the same Minebank Run USGS gage station and show similar means and ranges between the two datasets (Fig. S1).

### Statistical analyses

We used ANOVA to test for differences in chemistry (NO_3_^−^, DOC, and Cl^−^ concentrations) between groundwater and surface water and between pre-restoration and post-restoration periods at restored CVP and control Intervale. We used mixed-model ANOVA with the restoration treatment (pre and post restoration) as a fixed effect and time (sampling period) as a random effect. Separate ANOVAs were run for each constituent (NO_3_^−^, DOC, and Cl^−^). Separate models were constructed for the restored CVP and control Intervale reaches for each groundwater and surface water. We considered samples as independent replicates because of stream studies, including previous studies at Minebank Run (e.g. Groffman et al. 2005; Kaushal et al. 2008), showing high variability across features or microhabitats in streams, especially groundwater samples. This variability was likely due to a combination of stream geomorphology, hot spots of denitrification and organic carbon accumulation, and hydrology causing alternate flushing or legacy effects (Striz and Mayer 2008; Mayer et al. 2010b). To this point, we examined the spatial variability in NO_3_^−^, DOC, and Cl^−^ concentrations across stream features because we expected that chemical behavior would be driven by spatial and temporal dynamics affected by geomorphic differences. Therefore, we performed one-way ANOVA to compare NO_3_^−^, DOC, and Cl^−^ concentrations, respectively among stream features. Separate ANOVAs were run for each constituent for the pre-restoration and post-restoration periods, respectively. Tukey’s post-hoc tests were performed to compare means of the respective comparisons. We performed regression analyses on the USGS surface water samples over time. We developed separate regression models for the pre-restoration, construction, and post-restoration periods in order to capture trends across these distinct conditions. Data were analyzed using Systat 13.0 and SigmaPlot 14.0 software (https://systatsoftware.com).

## Results

### Evaluating evidence for failure of stream restoration features after 2008

**Fig. 4:**
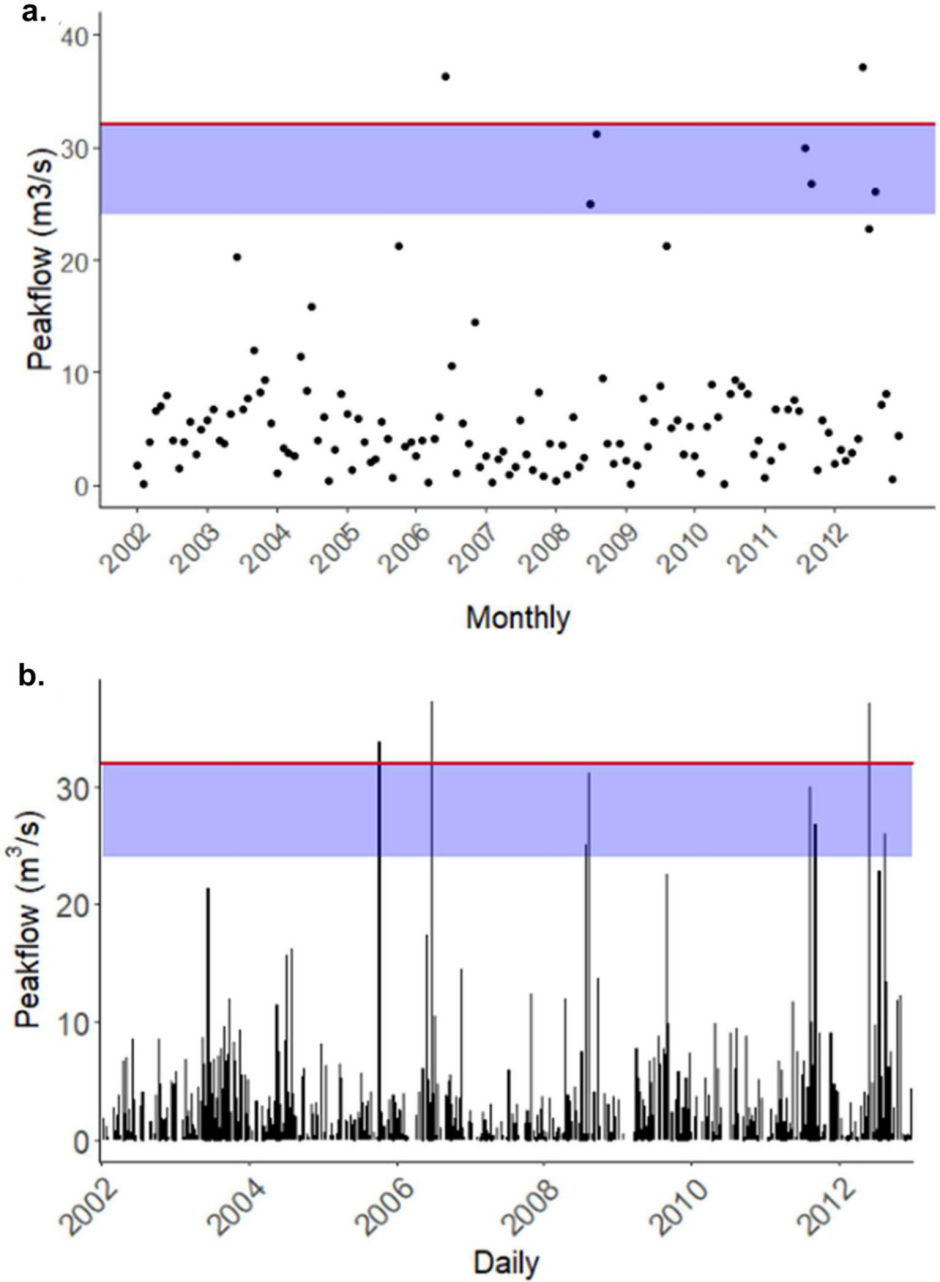
Monthly (**a**) and daily (**b**) peakflow, the discharge at which stormflow hydrograph is at its peak (m3/s) during the study period, calculated based on methods of Pennino et al. (2016). The red line is at 32 cubic meters per second (m^3^/s) and represents the 100-year recurrence interval discharge amount (Q100). The blue shaded region shows any peakflow values within 25% of Q100

Annual peakflow events were used to estimate the 100-year recurrence interval discharge (Q100) to determine the size of storm events that may be large enough to cause damage to stream channels. Using a bootstrapping approach and the recurrence interval, we calculated Q100 for Minebank Run to be 32 cubic meters per second (m^3^/s) (https://www.usgs.gov/special-topics/water-science-school/science/100-year-flood; Fig. 4). Hawley (2018) suggested that, to reduce the impacts of 100-year flood events, “Practitioners are encouraged to size their grade control armor to actually resist entrainment at a defensible recurrence interval (e.g., Q100 with an approximately 25%–50% factor of safety) and provide adequate thicknesses of the stone layers both vertically and tied into the banks laterally.” At Minebank Run, based on monthly peakflow events, we found two large storm events producing discharge above 32 m^3^/s in June 2006 and June 2012 (Fig. 4). There were five more monthly peakflow events within 25% of the Q100 that occurred in August 2008, August 2011, September 2011, August 2012, and July 2008 (Fig. 4). This corroborates the observed failure of many of the restoration features after 2008 (personal communication, Ed Doheny, USGS; Fig. 4).

### Precipitation trends and hydrologic response to stream-floodplain reconnection

**Fig. 5:**
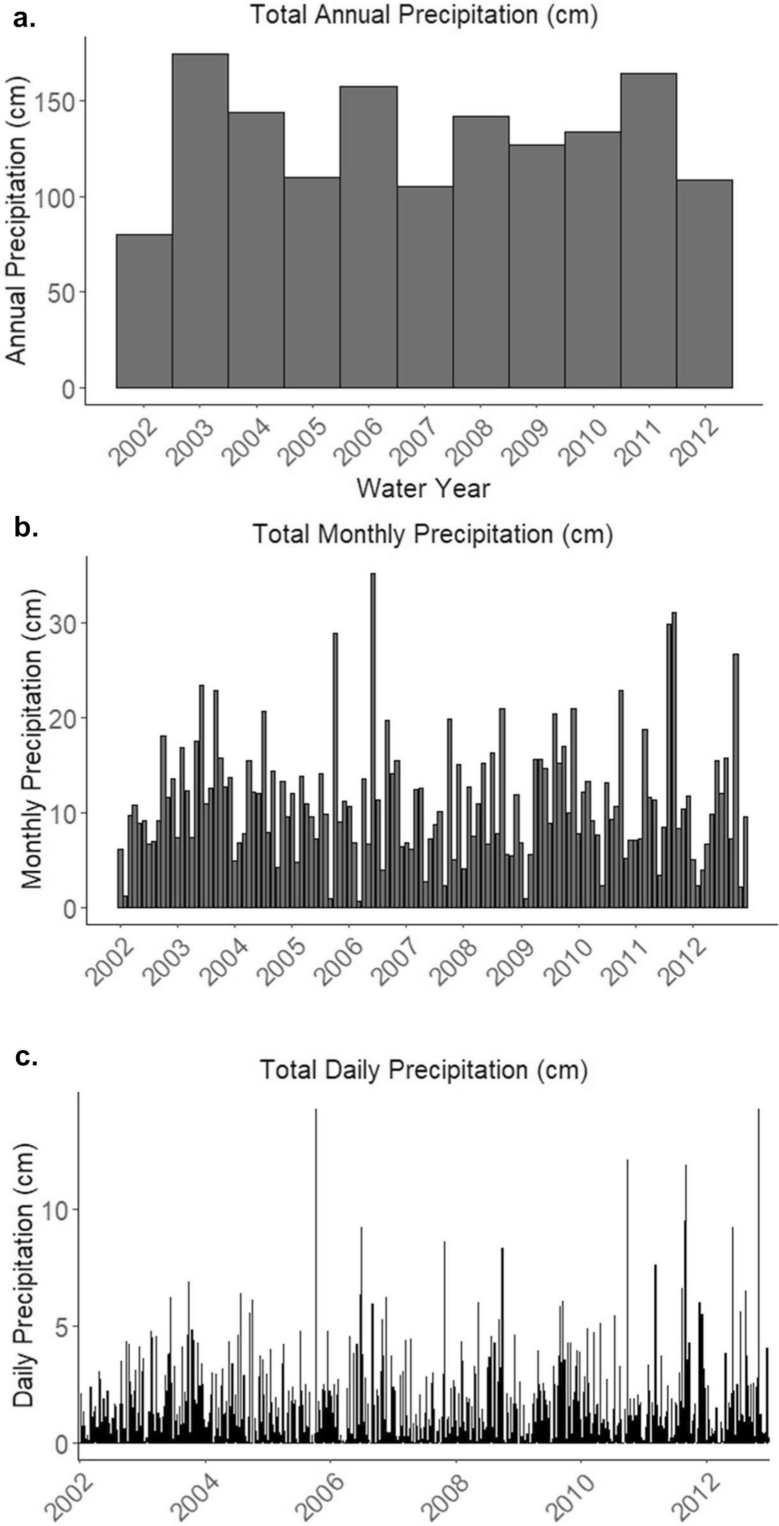
Total annual (**a**), monthly (**b**), and daily precipitation (**c**), downloaded from the PRISM Climate Group (https://prism.oregonstate.edu/explorer/; latitude: 39.4200/−76.5788)

**Fig. 6:**
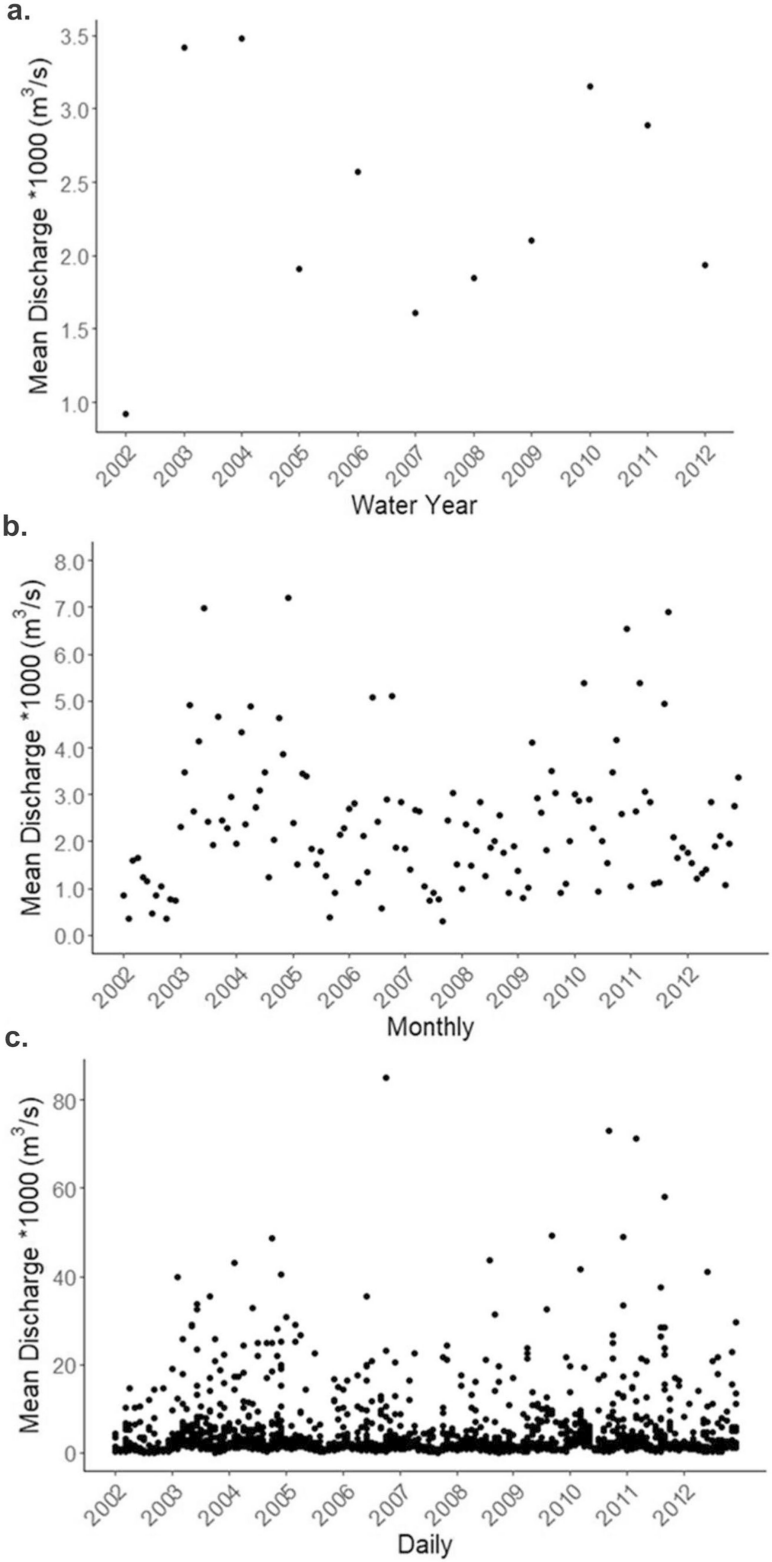
Mean annual (**a**), monthly (**b**), and daily (**c**) discharge at Minebank Run restored CVP reach (USGS gage 0158397967)

Annual precipitation during the study period ranged from 80 to 174 cm and was greatest in 2003 and 2011 and lowest in 2002 and 2007 (Fig. 5). Average annual discharge was highest in 2004 and 2003 and lowest in 2002 and 2007 (ranging from 900—3500 m^3^/s) (Fig. 6). The highest mean daily discharge days occurred on 27 Oct 2003, 8 Oct 2005, 13 Aug 2008, 11 Sept 2009, 26 Dec 2009, 30 Sept 2010, 10 March 2011, 7 September 2011, and 29 October 2012 (Fig. 6c). The top 10 peak flow events occurred on 12 June 2003, 8 Oct 2005, 25 June 2006, 23 July 2008, 13 Aug 2008, 28 Aug 2009, 14 Aug 2011, 7 Sept 2011, 1 June 2012, 14 Aug 2012 (Fig. 4).

**Fig. 7:**
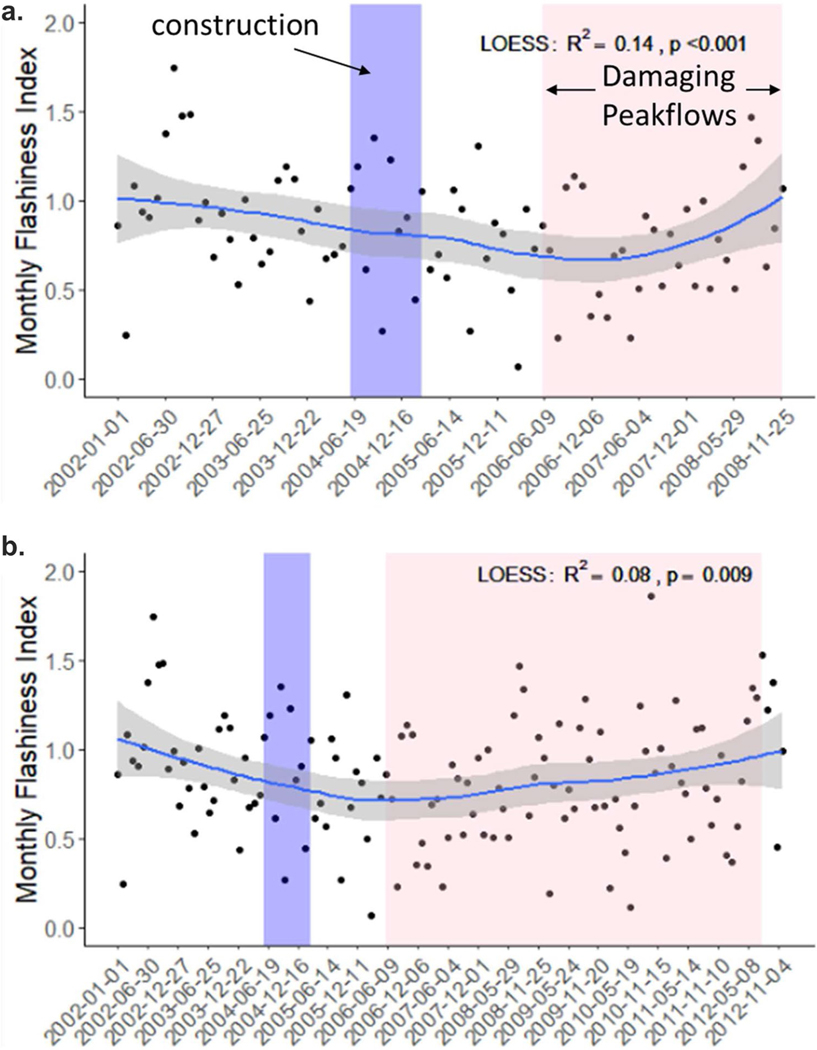
The average monthly Hydrologic Flashiness Index: (**a**) from 2002–2008 and (**b**) from 2002–2012. Two post-restoration periods are shown because there is evidence for failure of restoration features after 2008 which we attribute to damaging peak flows

**Fig. 8:**
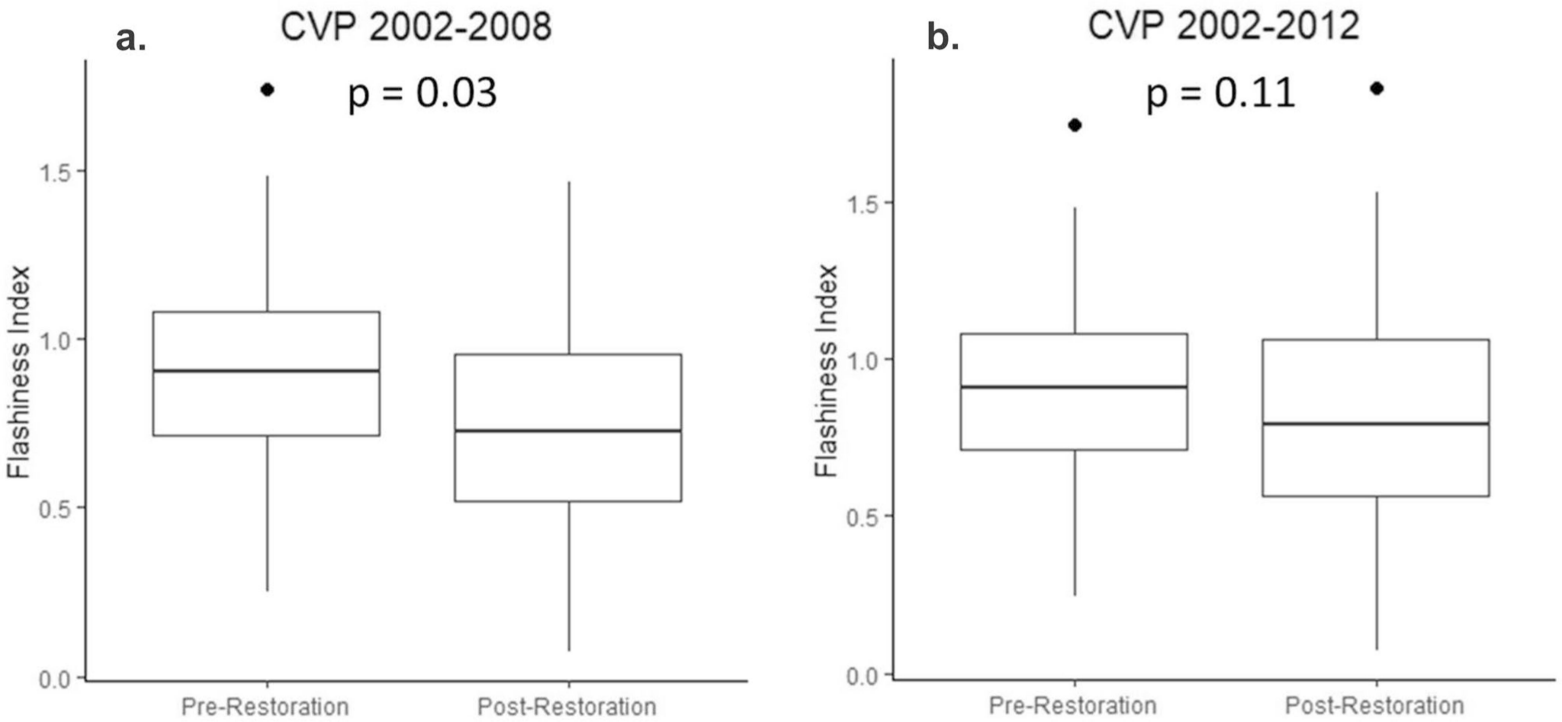
Hydrologic flashiness compared during pre- and post-restoration periods for (**a**) 2002–2008 and (**b**) 2002–2012. Two post-restoration periods are shown because there is evidence for failure of restoration features after 2008 which we attribute to damaging peak flows

A fundamental objective of the restoration was to reduce flashy flows during precipitation events. Monthly flashiness index, based on mean monthly discharge, decreased over time between 2002 and 2008 (Fig. 7a), but when extending the timeframe from 2002–2012, the flashiness index no longer shows a decline (Fig. 7b). Similarly, when specifically comparing the pre-restoration period (2002–2004) with the post restoration period of 2005–2008, there is a decline in the stream flashiness index (*p* = 0.03, Fig. 8a), but there is no difference when the post-restoration period is extended to 2012 (*p* = 0.11, Fig. 8b). By comparison, the flashiness of daily rainfall data (precipitation flashiness index) shows the opposite pattern with an increase in variability post-restoration (*p* = 0.05) in the 2002 to 2008 period and no significant change in the 2002 to 2012 period (*p* = 0.14, Figs. S2 and S3).

### Comparing groundwater vs surface water chemistry

**Fig. 9:**
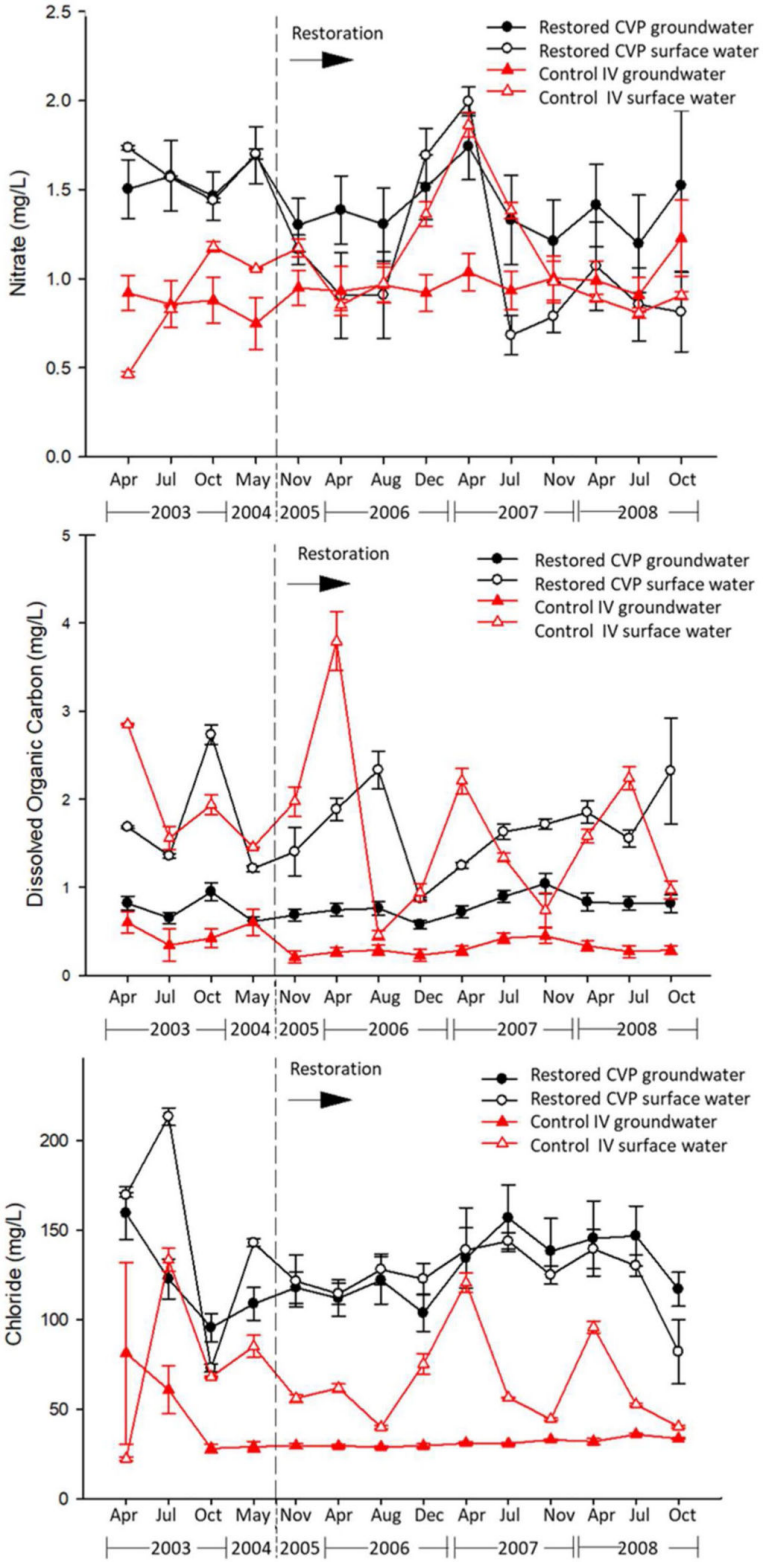
Minebank Run restored CVP and control Intervale (IV) reaches, NO_3_^−^, DOC, and Cl^−^ concentrations (mg/L) in groundwater and surface water. Time is not to scale

Groundwater NO_3_^−^ concentrations did not differ from surface water NO_3_^−^ concentrations during either the pre- or post-restoration periods at either the restored CVP or the control IV sites based on mixed-model ANOVA (*p* ≥ 0.09; Table 1; Fig. 9). In other words, groundwater resembled surface water at both reaches. DOC was always significantly higher in surface water than in groundwater both before and after restoration at both the control IV and the restored CVP reaches (*p* < 0.001; Table 1; Fig. 9), suggesting that DOC was transported in surface water without being stored in groundwater or because DOC was consumed while in the subsurface. Groundwater and surface water Cl^−^ concentrations were similar at the restored CVP reach both before and after restoration (*p* ≥ 0.07; Table 1; Fig. 9). However, at the control IV reach, surface water Cl^−^ was double that in the groundwater both before and after restoration (*p* < 0.001; Table 1), suggesting that local runoff events in the headwaters influenced surface water salt chemistry. Cl^−^ concentrations at the downstream restored CVP reach (Fig. 9) were chronically elevated compared to the upstream control IV reach due to effects of the I-695 Beltway, a major freeway that received heavy deicer salt inputs. Groundwater was a reservoir for salt loads (Cooper et al. 2014), leading to similar groundwater and surface water C l^−^ concentrations downstream (Fig. 9).

### Pre- and post-restoration patterns in groundwater and surface water chemistry

Groundwater NO_3_^−^ concentration was significantly lower after restoration at the restored CVP reach based on mixedmodel ANOVA (*F*_1, 422_ = 3.98; *p* = 0.05; Table 2; Fig. 9). The decline in groundwater NO_3_^−^ concentration after restoration based on the EPA data was overall about 15% from a mean of 1.56 mg/L (SE ± 0.08) to 1.33 mg/L (SE ± 0.06). Surface water NO_3_^−^ concentration also was lower after restoration at CVP (*F*_1, 44_ = 5.49; *p* = 0.02; Table 2; Fig. 9). The decline in surface water NO_3_^−^ concentration after restoration based on the EPA data was overall about 33% from a mean of 1.61 mg/L (SE ± 0.03) to 1.08 mg/L (SE ± 0.08). However, both surface water and groundwater NO_3_^−^ concentration at the control IV reach remained similar throughout the post-restoration period (*p* ≥ 0.18, Table 2; Fig. 9), suggesting that conditions upstream were not the cause of the downstream changes in NO_3_^−^ and that the effect on NO_3_^−^ at the CVP reach was due to the restoration.

Neither surface water nor groundwater DOC differed after restoration at the CVP reach (*p* ≥ 0.65; Table 2; Fig. 9). Likewise, surface water DOC at the control IV reach did not differ after the downstream restoration (*F*_1, 24_ = 0.37; *p* = 0.55; Table 2; Fig. 9). However, groundwater DOC was lower after the restoration at the control IV reach for reasons we were not able to ascertain (*F*_1, 181_ = 10.71; *p* = 0.001; Table 2; Fig. 9). This difference did not seem to propagate downstream to the restored reach suggesting that more local sources of organic matter were transported to the stream.

Neither groundwater nor surface water Cl^−^ concentrations at the restored CVP reach differed after the restoration (*p* ≥ 0.21; Table 2; Fig. 9). This suggests that the change in NO_3_^−^ concentration at CVP was due to biological control as Cl^−^ is conservative and is not altered significantly biologically (Mayer et al. 2010b). Likewise, neither groundwater nor surface water Cl^−^ concentrations at the control Intervale site reach differed after the restoration (*p* ≥ 0.26; Table 2; Fig. 9).

### Restoration effects on biweekly surface water chemical concentrations and fluxes

**Fig. 10:**
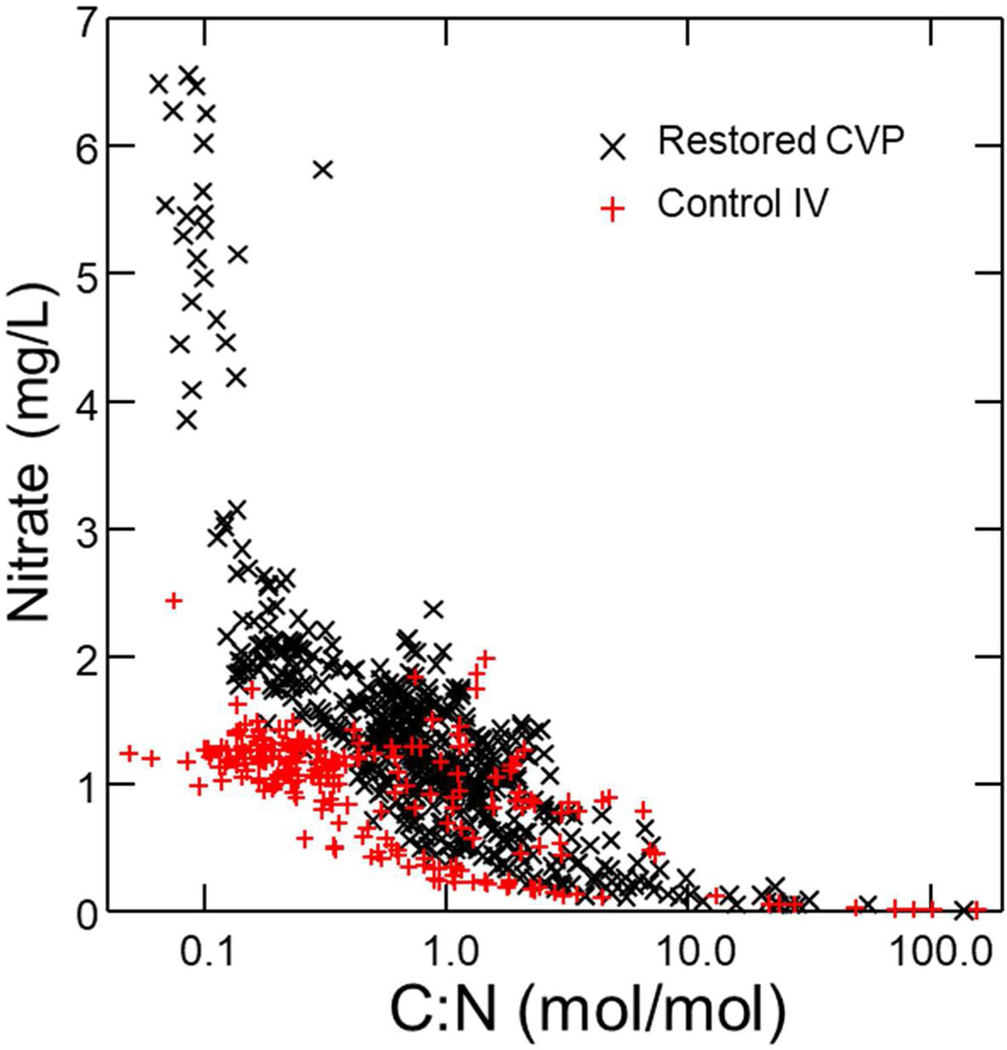
At Minebank Run, at both restored CVP reach and the control Intervale (IV) reach, groundwater NO_3_^−^ versus molar ratios of C:N exhibited a similar negative curvilinear relationship. This pattern is exhibited in aquatic ecosystems elsewhere (Taylor and Townsend 2010), and is indicative of microbial control of NO_3_^−^ dynamics

Based on linear regression, surface water NO_3_^−^ concentration in the intensive USGS surveys of surface water showed increasing trends prior to restoration and during construction at restored CVP (*p* ≤ 0.008; Table 3; Fig. 10). However, after restoration, NO_3_^−^ trends declined steadily (*p* < 0.001; Table 3; Fig. 10). Seasonal cycles were evident, with higher NO_3_^−^ observed in winter with maximum concentrations > 4 mg/L before restoration and > 2 mg/L even after restoration (Table 3) when temperatures and microbial and plant activity were lower, likely reducing uptake of NO_3_^−^. NO_3_^−^ was especially low (0.23 mg/L; Table 3) during a severe drought in 2002 and then rose concurrently with a rapid shift to a wet season in 2003 (Fig. 10). Similar trends occurred at control IV (Table 3; Fig. 10), suggesting that seasonal and interannual NO_3_^−^ cycles and runoff effects propagated downstream to the restored site. However, there are no comparable pre- and post-restoration comparisons of NO_3_^−^ at control IV because start of sampling coincided with the restoration construction period at CVP (Table 3).

**Fig. 11:**
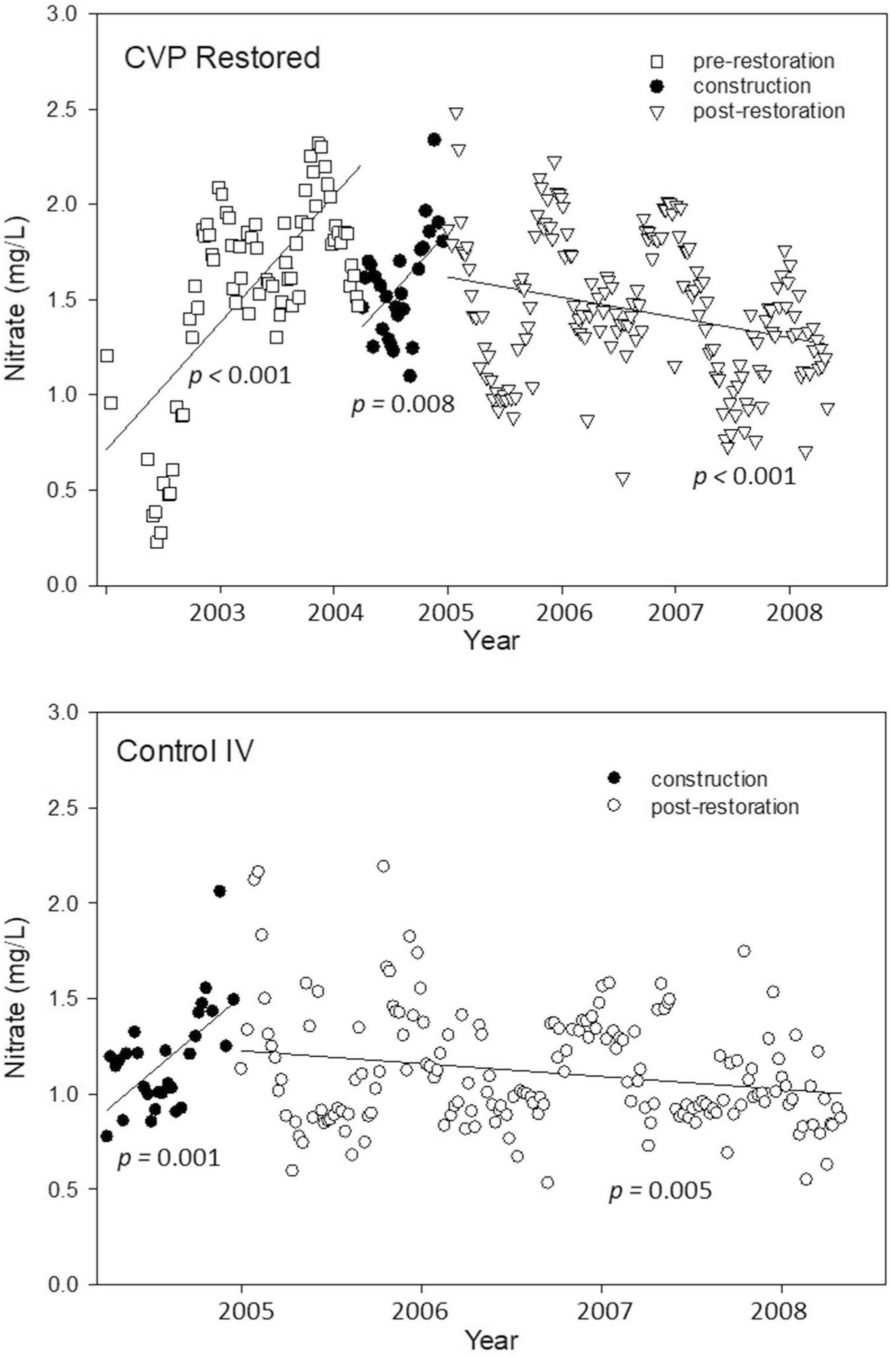
USGS bi-weekly Minebank Run surface water NO_3_^−^ concentrations (mg/L) at restored CVP and control Intervale (IV). NO_3_^−^ shows increasing trends prior to the restoration and during construction at the restored, downstream CVP reach. NO_3_^−^ trends decline steadily after the restoration. Seasonal cycles are evident and NO_3_^−^ was especially low during a severe drought in 2002 and then rose concurrently with a rapid shift to a wet season in 2003

Biweekly surface water sampling by USGS at restored CVP did not include regular Cl^−^ analysis. Instead, we relied on specific conductance as a surrogate measure of Cl^−^ which can reflect similar patterns as Cl^−^ flux (Cooper et al. 2014; Pennino et al. 2016). Like Cl^−^, specific conductance was chronically higher at the downstream restored CVP site than upstream at control IV (Fig. 11). Specific conductance was relatively variable, exhibiting peaks and outliers (Fig. 11). Based on linear regression, conductivity increased over time at restored CVP and control IV prior to restoration (*p* ≤ 0.05; Table 3). Post-restoration specific conductance trends were not significant at either restored CVP or control IV (*p* ≥ 0.11; Table 3).

### Relationship between NO_3_^−^ and DOC

**Fig. 12:**
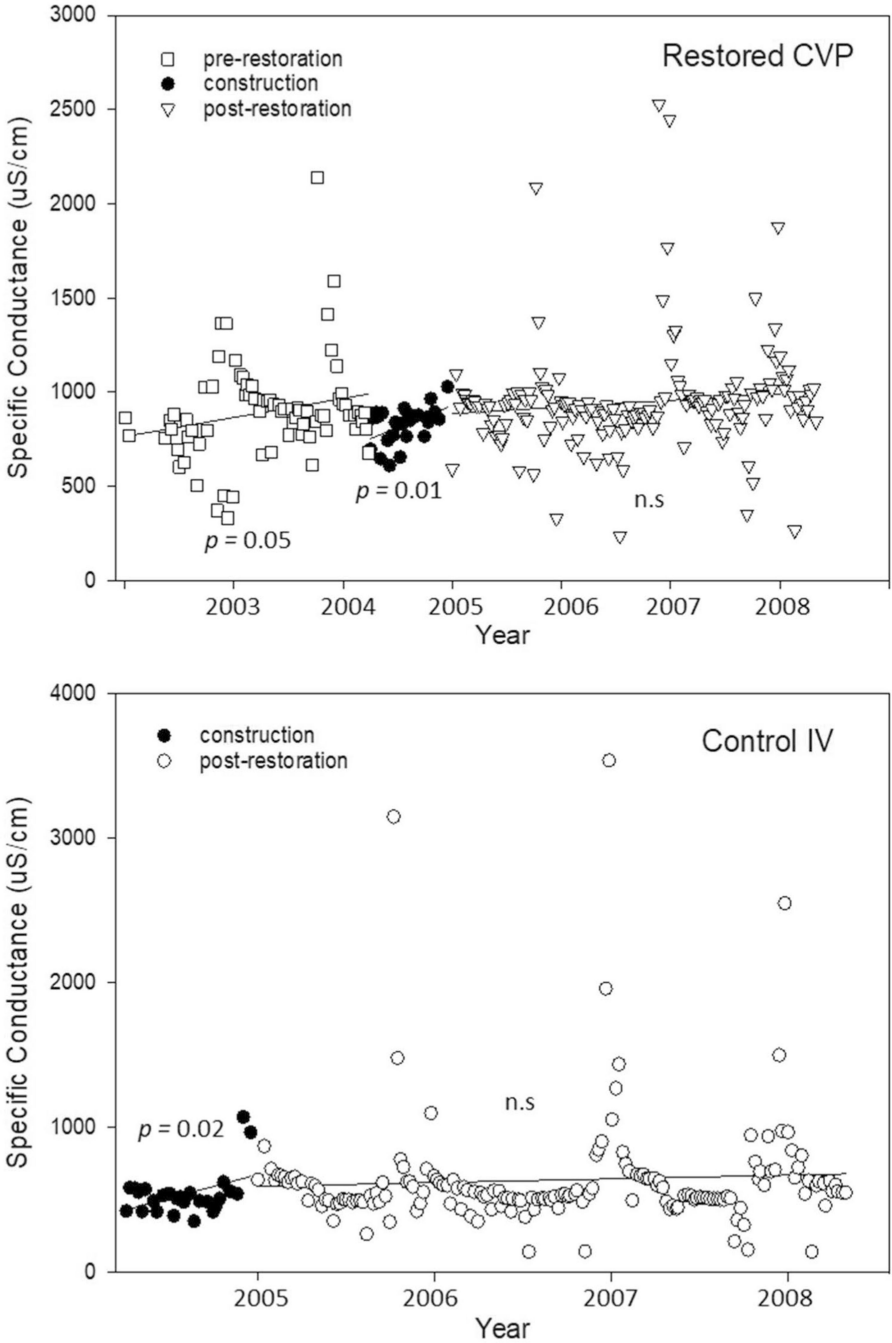
USGS bi-weekly Minebank Run surface water specific conductance (μS/cm) at restored CVP and control Intervale (IV). Surface water in samples collected bi-weekly at restored CVP reach. Specific conductance is generally higher at restored CVP than at the upstream control Intervale because of the influence of the I-695 beltway and associated inputs of road salts. Trends overall appear to be increasing with occasional extreme peaks from storm runoff suggesting that road salts and/or other ions are influencing water chemistry at Minebank Run

Ratios of NO_3_^−^ to C:N at both the restored CVP reach (*N* = 477; $\bar{X}$ ± SE: 2.15 ± 0.35) and the control IV reach (*N* = 232; $\bar{X}$ ± SE: 3.17 ± 0.97) showed the same negative curvilinear relationship (Fig. 12). Highest NO_3_^−^ concentrations were observed in piezometers where C:N ratio was lowest. Low NO_3_^−^ concentrations approached zero in groundwater samples where C:N was about 10:1.

### Chemistry response to channel geomorphology

NO_3_^−^, DOC, and Cl^−^ concentrations in groundwater differed among stream features at restored CVP reach before and after the restoration (*p* ≤ 0.05; Table 4). Before restoration, NO_3_^−^ was highest in cutbanks ($\bar{X}$ ± SE: 2.6 ± 0.3 mg/L; Table 4) followed by concentrations below the stream channel and in terrace features ($\bar{X}$ ± SE: 1.53 ± 0.06 mg/L and 1.51 ± 0.08 mg/L, respectively; Table 4). DOC was highest in the subsurface of the stream channel ($\bar{X}$ ± SE: 1.18 ± 0.08 mg/L; Table 4), likely as a function of transport and groundwater-surface water mixing. Cl^−^ was highest in groundwater of gravel bars associated with meander features and below the stream channel ($\bar{X}$ ± SE: 163.5 ± 11.3 mg/L and 135.0 ± 8.3 mg/L, respectively; Table 4) and lowest in cutbank features ($\bar{X}$ ± SE: 59.8 ± 6.3 mg/L; Table 4) where the stream was most disconnected.

After restoration, oxbows and rip rap structures became new features of the system. NO_3_^−^ was highest in oxbow features ($\bar{X}$ ± SE: 2.52 ± 0.25 mg/L; Table 4), suggesting higher retention of NO_3_^−^ at this floodplain reconnection feature (Harrison et al. 2012a). Cutbank features were mostly eliminated after the restoration, however NO_3_^−^ was relatively low for the few samples collected ($\bar{X}$ ± SE: 0.91 ± 0.09 mg/L; Table 4). NO_3_^−^ in the stream and at terrace features designed to connect the floodplain to the stream channel were similar (Table 4), suggesting again that the stream and terraced features were hydrologically connected. DOC remained highest ($\bar{X}$ and SE: 1.23 ± 0.06 mg/L; Table 4) below the stream channel perhaps owing to higher transport of incoming organic matter mixing in the subsurface. Chloride was highest at gravel bars and below the stream channel ($\bar{X}$ ± SE: 212.3 ± 13.9 and 128.5 ± 3.3 mg/L, respectively; Table 4), suggesting enhanced Cl^−^ storage in these features. Chloride concentration was similar among oxbows, riprap, and terrace features (Table 4).

### Trends in NO_3_^−^ and TN fluxes from bi-weekly monitoring at Minebank Run

**Fig. 13:**
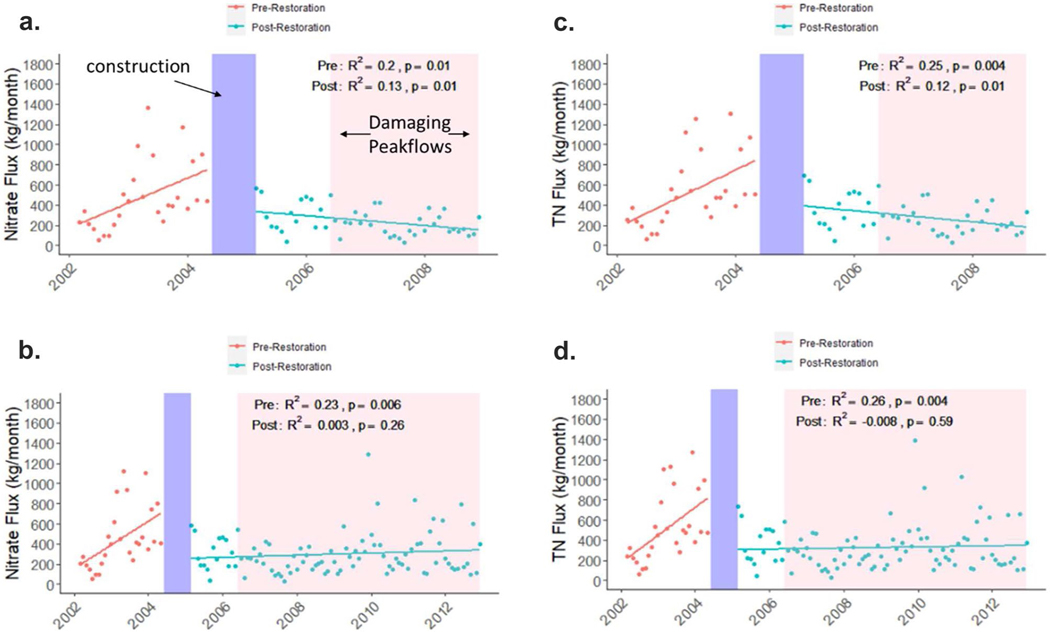
Trends in NO_3_^−^ flux (kg/month) compared during pre- and post-restoration periods for (**a**) 2002–2008 and (**b**) 2002–2012. Trends in TN flux (kg/month) compared during pre- and post-restoration periods for (**c**) 2002–2008 and (**d**) 2002–2012. The blue regions represent the timeframe for the restoration and the pink region represents the timeframe for high peakflows, sufficient to cause geomorphic changes. Two post-restoration periods are shown because there is evidence for failure of restoration features after 2008 which we attribute to damaging peak flows

**Fig. 14:**
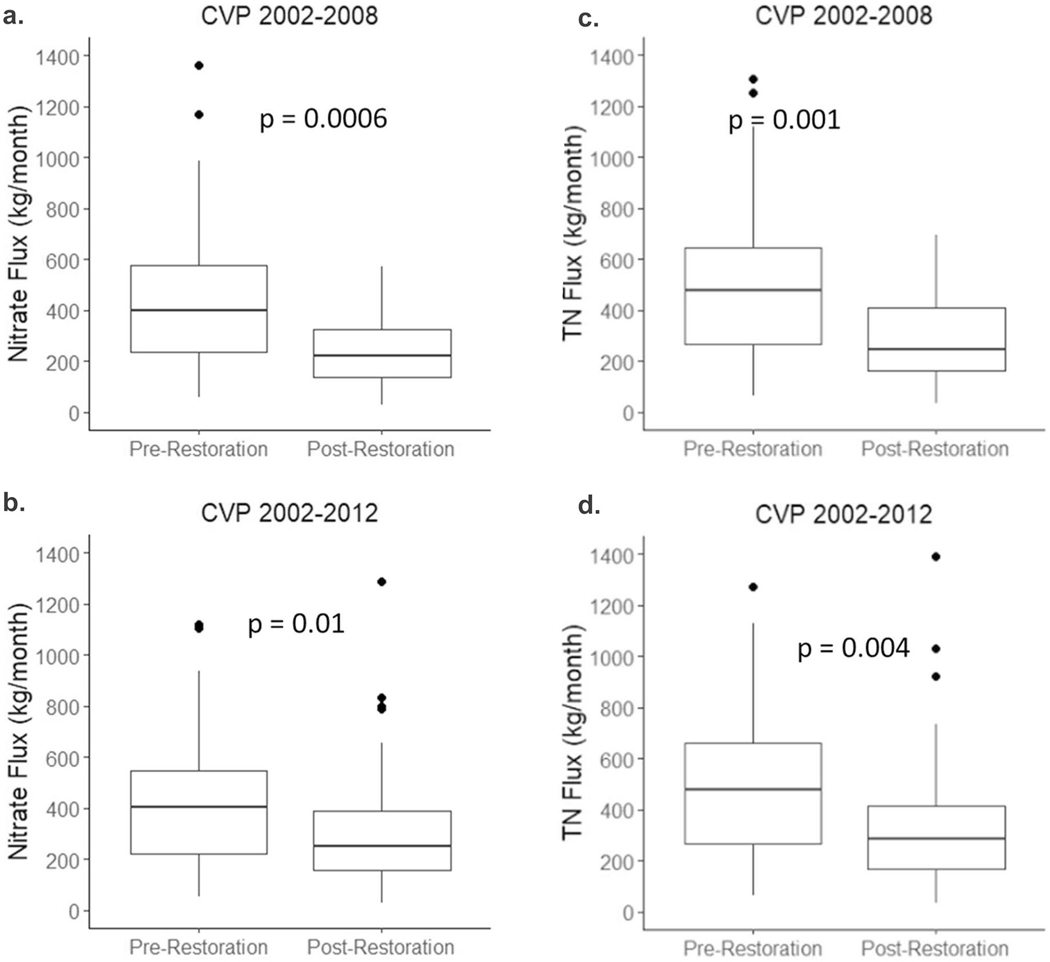
Pre- and post-restoration box plots of monthly NO_3_^−^ flux (kg/month) at Minebank Run for (**a**) 2002–2008 and (**b**) 2002–2012. Pre- and post-restoration box plots of monthly TN flux (kg/month) at Minebank Run for (**c**) 2002–2008 and (**d**) 2002–2012. Two post-restoration periods are shown because there is evidence for failure of restoration features after 2008 which we attribute to damaging peak flows

**Fig. 15:**
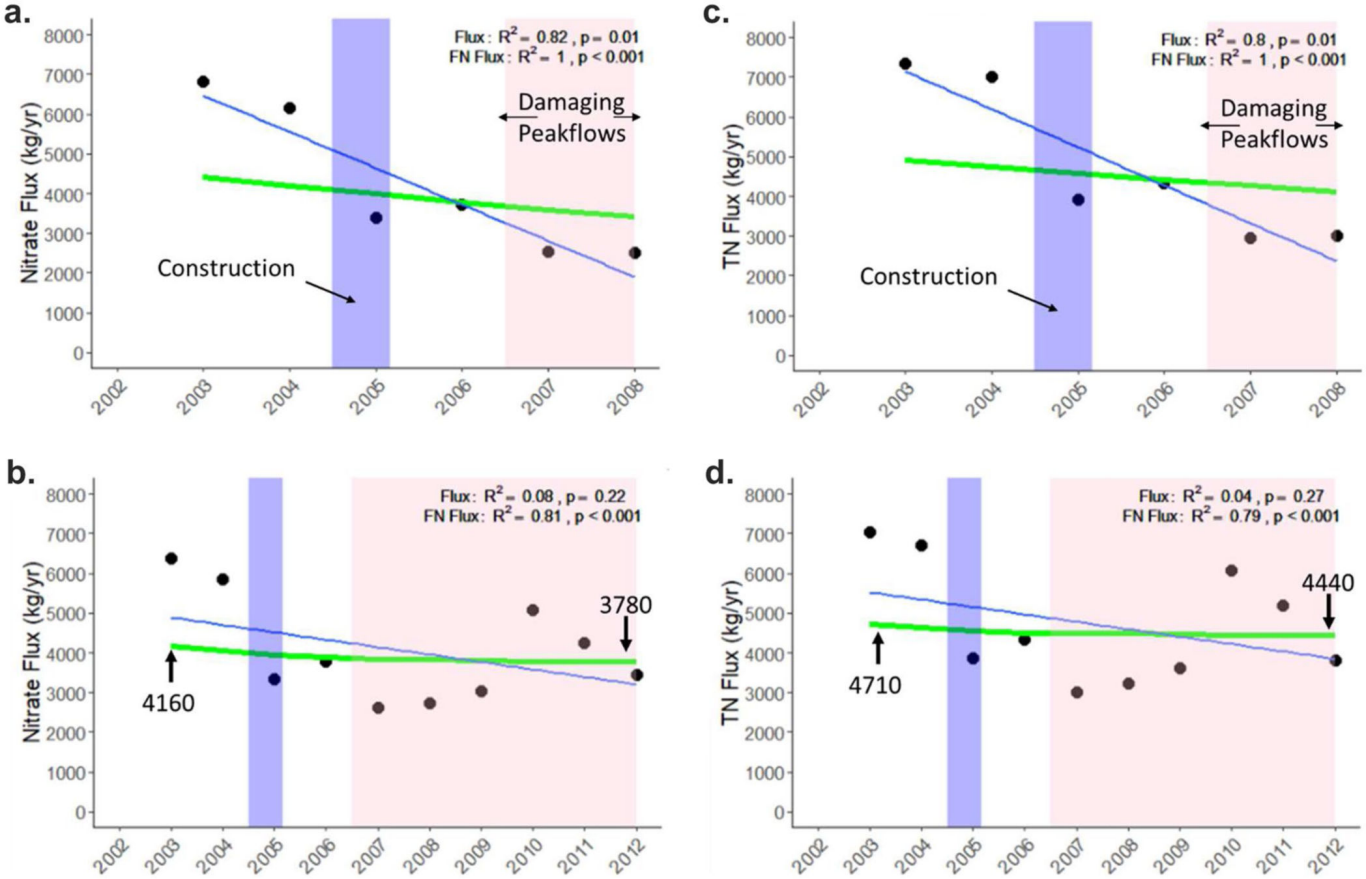
Annual average NO_3_^−^ flux (black dots and blue trendline) and flow normalized (FN; green) flux at Minebank Run for the (**a**) 2002–2008 and (**b**) 2002–2012. Annual average total nitrogen (TN) flux (black dots and blue trendline) and flow normalized (FN; green) flux at Minebank Run for the (**c**) 2002–2008 and (**d**) 2002–2012. The blue regions represent the timeframe for the restoration and the pink region represents the timeframe for high peakflows, sufficient to cause geomorphic changes. Two post-restoration periods are shown because there is evidence for failure of restoration features after 2008 which we attribute to damaging peak flows

Monthly NO_3_^−^ flux estimates showed an increasing trend pre-restoration (*R*^2^ = 0.2, *p* = 0.01), and a decreasing trend post-restoration from 2005–2008 (*R*^2^ = 0.13, *p* = 0.01; Fig. 13a), but there was no change in flux post-restoration from 2005–2012 (*R*^2^ = 0.003, *p* = 0.26; Fig. 13b). A similar pattern is also seen for TN flux (Fig. 13c, d). When comparing the monthly pre- and post-restoration flux values, there is a significant decline in flux in the first three years post-restoration (*p* < 0.001; Fig. 14a) and in the first seven years post-restoration (*p* = 0.01; Fig. 14b). The same pattern is found for TN flux (Fig. 14c, d). When looking at trends in annual flux and FN flux, for both NO_3_^−^ and TN, there is a steady decline in both from 2002–2008 (*p* = 0.01 and *p* < 0.001, respectively; Fig. 15a, c). For the 2002–2012 period there is a less pronounced decline in FN NO_3_^−^ and TN flux (*p* < 0.001), but no significant decline in annual flux (*p* = 0.22 and *p* = 0.27, respectively; Fig. 15b, d), perhaps due to several in-stream constructed features eroding and damaged after storms and the oxbow wetland filling in beginning around 2008.

**Fig. 16:**
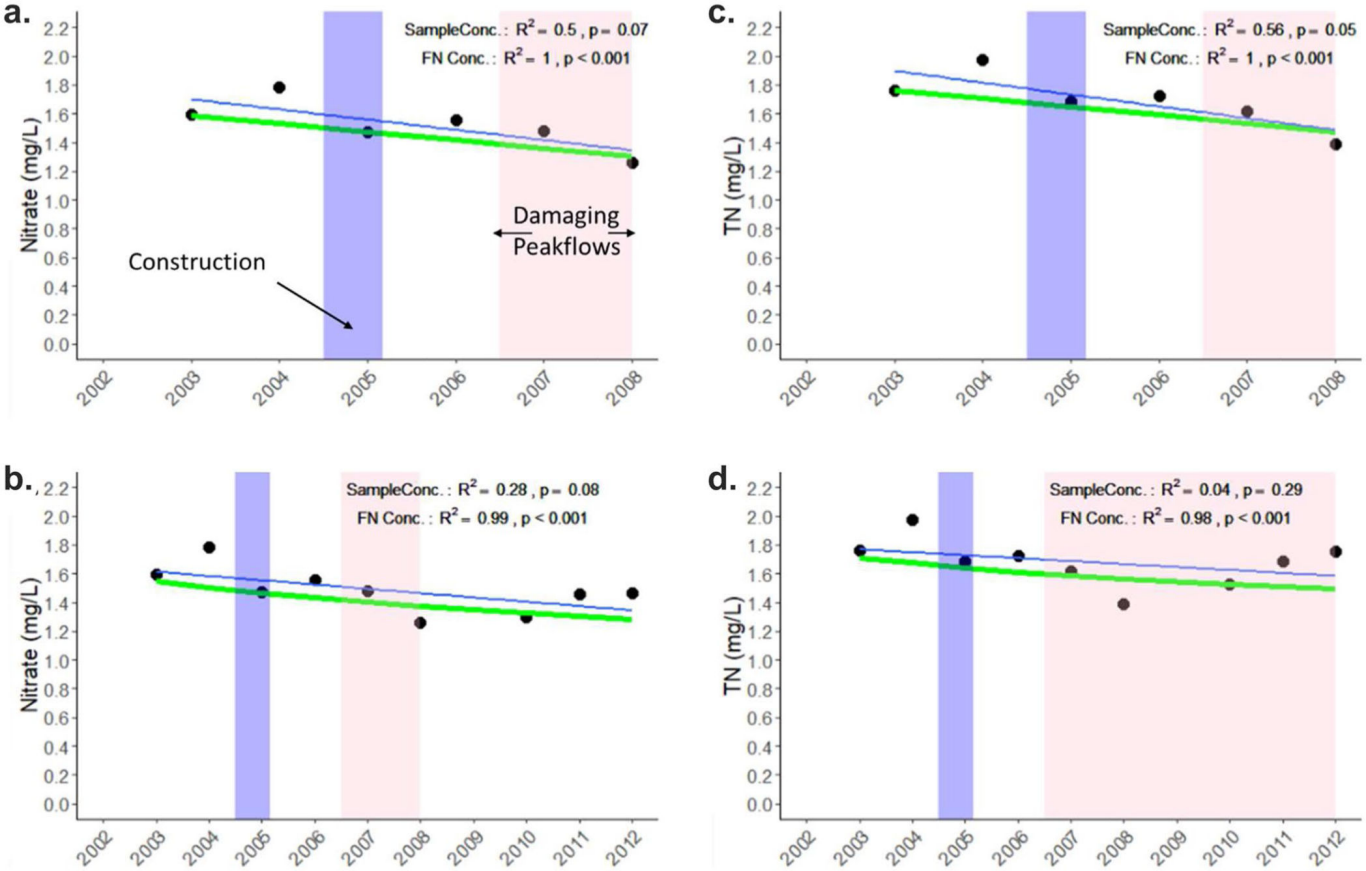
Annual mean NO_3_^−^ concentration (black dots and blue trendline) and flow normalized concentration trends (green) for the (**a**) 2002–2008 and (**b**) 2002–2012. Annual mean total nitrogen (TN) concentration (black dots and blue trendline) and flow normalized concentration trends (green) for the (**c**) 2002–2008 and (**d**) 2002–2012. The blue regions represent the timeframe for the restoration and the pink region represents the timeframe for high peakflows, sufficient to cause geomorphic changes. Two post-restoration periods are shown because there is evidence for failure of restoration features after 2008 which we attribute to damaging peak flows

**Fig. 17:**
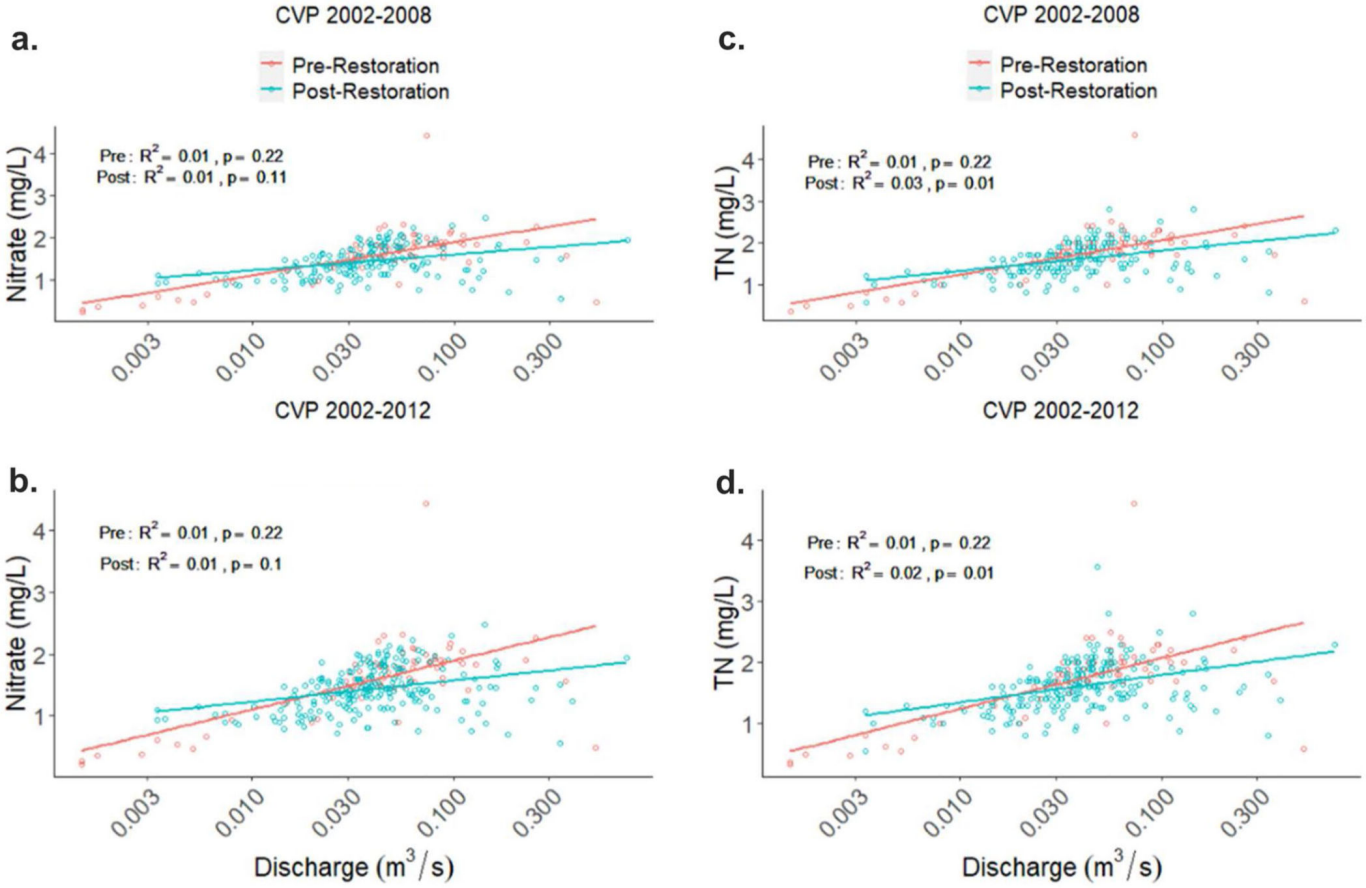
Nitrate (NO_3_^−^) concentration vs discharge relationship during pre- and post-restoration periods for (**a**) 2002–2008 and (**b**) 2002–2012. Total nitrogen (TN) concentration vs. discharge relationship during pre- and post-restoration periods for (**c**) 2002–2008 and (**d**) 2002–2012. Two post-restoration periods are shown because there is evidence for failure of restoration features after 2008 which we attribute to damaging peak flows

The EGRET package was also used to calculate mean annual flow normalized (FN) concentrations and we found that FN NO_3_^−^ and TN concentrations declined over the 2003–2008 period (*R*^2^ = 1.0, *p* < 0.001; Fig. 16a) and the 2003–2012 period (*R*^2^ = 0.99, *p* < 0.001; Fig. 16b). However, we found no relationship between NO_3_^−^ concentration and discharge for either the 2003–2008 or 2003–2012 time periods (*p* ≥ 0.11). Only TN post-restoration had a significantly positive relationship with discharge in both the 2002–2008 and 2002–2012 periods (Fig. 17c, d, p = 0.01). This is likely because discharge is more closely linked to particulate N than dissolved N forms.

## Discussion

Chemistry trends at Minebank Run suggest that stream restoration involving floodplain reconnection has the potential to reduce NO_3_^−^ concentration and NO_3_^−^ flux at a reach scale, provided that the stream restoration remains stable and the geomorphic features that contribute to the processing and transformation of N remain intact. Based on the BACI design, there was no change in NO_3_^−^ concentration in the control IV reach after restoration while there was significant reduction in NO_3_^−^ concentration in the impacted (restored CVP) reach, suggesting that the effect was due to restoration. Furthermore, the lower concentrations of NO_3_^−^ in surface water and groundwater after restoration, while no coinciding change in conservative Cl^−^ and/or specific conductivity after restoration, suggests that the decreases in NO_3_^−^ are a function of biological uptake or denitrification (Mayer et al. 2010b) and not a function of simple dilution effects (Altman and Parizek 1995). Previous work at Minebank Run has shown that when stream channels are restored to allow overbank flow, denitrification rates are higher than where the channel flows through high, incised banks (Kaushal et al. 2008; Klocker et al. 2009). Below, we discuss the impacts of stream restoration on long-term patterns and processes of N transport and retention.

### Stream-floodplain reconnection (and other management activities) influences long-term N transport and retention

Our study demonstrated that floodplain reconnection was an effective restoration approach for reducing NO_3_^−^ concentration and flux in an urban stream. Both groundwater and surface water NO_3_^−^ decreased at restored CVP after the restoration despite no change in NO_3_^−^ concentration at the control site. Furthermore, NO_3_^−^ flux continued to trend downward after the restoration. These results suggest that the restoration was effective at a reach scale in reducing NO_3_^−^. Despite immediate positive effects of the restoration on nitrogen transformation, long-term flux measures of N showed that the restoration approach was unable to remain effective after several years due to high flow events destroying much of the restoration features.

While atmospheric N declines in the Chesapeake Bay area (Lovett et al. 2000; Linker et al. 2013), have contributed to corresponding declines in stream N (Eshleman et al. 2013), it is unlikely that such declines contributed to changes in chemistry at the restored reach of Minebank Run. The lack of observable declines at the control IV reach over time suggests that any probable reduction in atmospheric inputs had a much lesser effect on NO_3_^−^ flux at Minebank Run than did restoration. Furthermore, terrestrial inputs are unlikely to have declined over time given the consistent urbanization pressure. Restoration at Minebank Run appears to have made an impact on NO_3_^−^ flux through improved groundwater-surface water interaction and the initial reconnection of the floodplain to the channel. However, with numerous structural failures appearing along the restored reach (see Fig. S4a and b), long-term efficacy is in question. While restoration effects suggest that such management was effective at stabilizing current N inputs for the period of study, significant future reductions in NO_3_^−^ at Minebank Run will likely require additional management efforts and/or reductions in watershed nutrient inputs.

### Evidence that altered stream morphology enhances N transformation by increasing retention times in stream features and floodplains

The similarity of groundwater and surface water NO_3_^−^ and Cl^−^ at the restored CVP reach suggests mixing of groundwater and surface water. NO_3_^−^ and Cl^−^ patterns among stream restoration features varied before and after restoration suggesting that some features were more retentive of N. The oxbows created by the restoration had higher NO_3_^−^ concentrations perhaps because they were designed to retain stormwater runoff (Harrison et al. 2014). However, these features promoted high rates of denitrification (Harrison et al. 2011), demonstrating that such wetlands have the potential to reduce NO_3_^−^ in urban watersheds. The change in the proportions of riffles, runs, and pools may also have influenced the uptake of NO_3_^−^. The proportion of pools, which were found to have higher sediment denitrification potential than riffles (Harrison et al. 2012b), increased after restoration relative to riffles (Doheny et al. 2012). Overall, this suggests that altering stream geomorphology features may enhance N transformation by maintaining anaerobic conditions and microbial activity that stimulate denitrification (Harrison et al. 2012b).

N sink and source dynamics vary among geomorphic structures depending on the influence of geomorphology on hydrology and subsequent microbial activity (Munn and Meyer 1990; Jones and Holmes 1996; Kemp and Dodds 2002; Fisher et al. 2005). There was considerable change in channel morphology (e.g. increased proportions of riffles, run, pools, reduced sinuosity, reduced bank elevation and slope, reduced incision). Collectively, these changes influenced hydrology (i.e. flashiness), and thus nutrient transformation processes. Furthermore, boundary sheer stress was reduced, thereby reducing the erosive forces on the channel bed and banks of the stream (Doheny et al. 2007, 2012). Boundary shear stress and mean velocity values for Minebank Run were generally greater than non-urban B or C channel types (Doheny et al. 2012; Doheny and Baker 2018). The slope of the shear stress regression line for Minebank Run is considerably flatter than those for non-urban streams suggesting that small changes in mean velocity and discharge result in large changes in boundary shear stress and susceptibility to erosion (Doheny and Baker 2018), which may be an underlying reason for structural failures at Minebank Run. This could have resulted in observed changes in the slope of the NO_3_^−^ flux trend over the long term due to subsequent loss of N transformation functionality. Thus, reducing shear stress, flashiness, and scouring of organic matter, and instead, controlling flows to facilitate formation of debris dams may improve denitrification at Minebank Run and other similar urban streams (Groffman et al. 2005; Harrison et al. 2012b), but changes over time due to failures in geomorphic stability may have consequences on maintaining water quality functions.

Altered stream morphology after restoration also likely contributed to a reduction in hydrologic flashiness at Minebank Run. A previous study at Minebank Run showed that restoration lessened the positive relationship between precipitation and daily peak discharge, suggesting that restoration reduced overall flashiness of the system (Pennino et al. 2016). In another Minebank Run study, channel depth in the restored CVP reach was unchanged by restoration, however, channel width increased, and, consequently, cross-sectional area increased which caused a proportional decrease in mean flow velocity for comparable discharges (Doheny et al. 2012). Increased stream surface area has been shown across stream restoration approaches to improve nutrient retention (Newcomer-Johnson et al. 2016; Grant et al. 2018). Therefore, the increase in cross-sectional area may have contributed to the reduction in NO_3_^−^ flux that we observed in this study.

### Enhanced hydrologic connectivity in stream restoration is linked to N retention and transformation

The decline in post-restoration NO_3_^−^ concentrations and flux is supported by previous studies at Minebank Run showing that restored low “connected” banks consistently had higher in situ denitrification rates (Kaushal et al. 2008; Mayer et al. 2013). The restoration dramatically altered stream bed elevations in many places along the reach (Doheny et al. 2012) likely leading to improved connection between the stream channel and banks (Striz and Mayer 2008). Wider channel width and decreased channel incision may have increased hydrologic connectivity between groundwater and surface water and, thereby, affected denitrification rates (Groffman et al 2002, 2003). Changes in channel morphology to a mix of runs, riffles, and pools may have also enhanced N uptake by increasing the turbulence along bioreactive microbial films that control denitrification (Grant et al. 2018). The step-pool sequences, stream barbs, and meanders such as those in low connected reaches have been shown to increase hydrologic residence times and nitrogen retention in transient storage zones at the riparian-stream interface (Kasahara and Hill 2006) relative to straighter runs (Hill et al. 1998; Gücker and Boechat 2004). Hydrologic flowpaths in the more connected banks may have fostered higher denitrification rates as was demonstrated in previous work at Minebank Run using conservative tracer injections showing that lateral groundwater inputs along the riparian-stream interface can be substantial (Klocker et al 2009).

### Additional importance of organic matter and potential role for enhancing N transformation

DOC showed no change after restoration at CVP. However, DOC and NO_3_^−^ are linked. NO_3_^−^ declined with increasing C:N in a negative curvilinear trend suggesting that N transformation is C limited, a result consistent with previous studies at Minebank Run (Mayer et al. 2010b) as well as patterns across ecosystem types showing DOC limitation of denitrification (Taylor and Townsend 2010). Therefore, factors that influence accumulation and processing of C are strong regulators of N dynamics in streams. NO_3_^−^ approached zero where C:N was about 10:1 suggesting that management of stream N will be enhanced if riparian zones are kept intact to provide leaf litter as a C source (Wood et al., this issue), or where C is available from the rooting zone via exudates and decomposition (Gift et al. 2010), and where organic matter can be retained in the stream for denitrification (Groffman et al. 2005; Lazar et al. 2014). Current protocols for restoring streams in the Chesapeake Bay to manage N emphasizes engineering floodplains to maintain a zone of rooted vegetation to maximize NO_3_^−^ transformation potential (Urban Stormwater Work Group 2020).

Our observation of consistently higher DOC in surface water versus groundwater at Minebank Run suggests that organic matter does not mix in the subsurface and/or is consumed rapidly. Previous work has shown that DOC concentration is highest in surface water and declines with depth in the channel (Mayer et al. 2010b) likely due to limited water influx into the hyporheic zone. Organic matter is more abundant in upper root zone layers (Gift et al. 2010) and declines with depth in riparian soils (Groffman et al. 2002). Furthermore, particulate matter may be less likely to remain entrenched in interstitial zones or persist in debris and leaf packs in flashy streams (Groffman et al. 2005), thereby limiting the availability of organic matter in the subsurface. Because DOC is a critical limit to denitrification, restoration techniques designed to supply DOC more effectively to hyporheic zones and floodplains can optimize N removal (Newcomer-Johnson et al. 2016; Duan et al. 2019).

Previous research at Minebank Run showed that denitrification enzyme activity (DEA) and microbial biomass C were both higher in hyporheic sediments than in deep floodplain sediments suggesting that the hyporheic zone is responding to and processing C and NO_3_^−^ from upstream and/or riparian sources (Mayer et al. 2010b). These results also suggest that restoration that increases carbon availability in sediments could enhance denitrification capacity in stream ecosystems. At Minebank Run and other streams in the Baltimore area, denitrification potential was highest in organic debris dams and other features high in organic matter (Groffman et al. 2005). None of the stream features in Minebank Run were hot spots of high organic matter accumulation probably because flashy stream flows frequently wash debris from the channel and downstream. Also, pools in both the restored and unrestored reaches of Minebank Run had lower denitrification enzyme activity than pools from other streams in the Baltimore area (Groffman et al. 2005). Strong positive relationships at Minebank Run between root biomass and soil organic matter, and between soil organic matter and denitrification potential, suggests that deep rooted vegetation may be particularly important for maintaining an active denitrification zone in restored riparian zones (Gift et al. 2010). However, stream restoration approaches that improve hydrologic connectivity between hyporheic zones and floodplains (so that organic matter reaches subsurface zones where there is low DO and adequate NO_3_^−^ for anaerobic activity) is key to enhancing denitrification.

### Reductions in NO_3_^−^ and TN flux and flow normalized concentrations post-restoration

NO_3_^−^ and TN flux showed a significant decline in the 2005–2008 post restoration period compared to the 2002–2004 pre-restoration period, but the absolute NO_3_^−^, TN, and FN fluxes increased slightly during the 2010–2012 post-restoration period. Mean annual concentrations and flow normalized concentrations continued to decline during the full post-restoration period 2005–2012. There are several possible explanations for the increase in NO_3_^−^ flux in 2010–2012 including greater mean annual discharge and the observed failure of many of the restoration features after 2008. There is evidence that damage occurred to the restored features by 2013 and, based on the flow record, there were likely damaging peaks in 2009, 2011, 2012 (personal communication, Ed Doheny, USGS; Fig. 4). There were peak flow events > 30 m^3^/s at least three times between August of 2011 and June of 2013 (Fig. 4). In 2008, there was evidence of erosion at the toe of the banks in some of the cross sections, in 2011–2012, particularly August through September 2011, which was a wet period due to tropical storms. Some restoration features were degrading and many of the cut banks had re-established themselves. Based on observations by USGS (Ed Doheny, personal communication), by 2013, the area downstream of the sampling wells at restored CVP looked like degraded pre-restoration conditions with cut banks re-established, cross vanes buried and damaged, rock weirs collapsed, and without maintenance of the grade control of the channel bed (Fig. S4a and b). By 2016, the restoration at CVP had largely reverted to a pre-restoration condition where incised banks had re-appeared and the stream was no longer connected to the floodplain. Additionally, the large oxbow feature created in 2004 to accept excess stormflow upstream of the USGS gage, had filled with sediment by 2016, and perhaps as early as 2010, significantly reducing its original depth of about 1.5 m (Harrison et al. 2012a), and was no longer functioning to accept stormflow connected to the channel (personal communication, Ed Doheny).

The failure of restoration features after 2008 may have not only contributed to less NO_3_^−^ removal due to less biogeochemical processing, but also less attenuation of the discharge (Woltemade and Potter 1994; Hammersmark et al. 2008; Sholtes and Doyle 2011; Jacobson et al. 2015). This may partly explain why discharge was higher in 2010–2012 even though precipitation did not differ between the periods of 2005–2008 and 2010–2012 (Fig. 5). Magnitude and intensity of individual storms may also have been a factor. Also, less infiltration and less attenuation of peak discharge may have occurred after the oxbow feature was dysfunctional and the stream was no longer connected to the floodplain (Fink and Mitsch 2007; Hudson et al. 2012; Harrison et al. 2014; Palmer et al. 2014). There may have been less removal of N from the stream compared to when the oxbows were at peak function in the first years after restoration (Harrison et al. 2014). Consequently, the increase in NO_3_^−^ flux in 2010–2012 may be explained by the higher discharge and lesser biogeochemical processing due to the geomorphic failure of the restoration features and it may explain why stream flashiness, which initially declined after restoration, increased by 2008. Similarly, Thompson et al. (2018) observed little reduction in hydrologic flashiness at a reach scale after restoration at a stream in Maryland, USA.

Despite geomorphic failure of restoration features, the decline of flow normalized NO_3_^−^ concentrations over time indicate that the stream was still able to continue removing NO_3_^−^ through biogeochemical processing. Also, the lower slope of the NO_3_^−^ and TN concentration-to-discharge relationships during post-restoration compared to pre-restoration, indicates that, even at higher flows, N concentrations were lower after the restoration, which confirms that the restoration improved biogeochemical NO_3_^−^ uptake activity across a range of flows.

## Conclusions and recommendations

Our studies support the idea that in-stream processes and hydrologic connectivity between the stream channel and subsurface zones may influence N processing in urban streams (Craig et al. 2008, Kaushal et al. 2008). Restoration activities focused on increasing hydrologic connectivity in riparian zones may enhance denitrification rates by increasing soil organic C availability and altering hydrologic flowpaths (e.g. Fennessy and Cronk 1997; Groffman et al. 2003; Boulton 2007; Mayer et al. 2007). Because riparian soils, geomorphology, hydrologic flowpaths, N loads, and geology all play roles in explaining variation in denitrification rates (e.g. Alexander et al. 2000; Stanley and Doyle 2002; Groffman and Crawford 2003; Gücker and Boechat 2004; Wollheim et al. 2005; Mulholland et al. 2008), all of these factors should be considered and further evaluated in the efficacy of restoration designs aimed at increasing both denitrification rates and mass removal of NO_3_^−^ in riparian zones.

Restoration practices that improve hydrologic connectivity between the stream and the riparian zone can increase NO_3_^−^ removal. Yet, more work is necessary to better quantify the effectiveness of stream restoration practices under various applications and conditions and over time (Stanley and Doyle 2002; Kasahara and Hill 2006; Bukaveckas 2007; Roberts et al. 2007). For example, little is known about how N removal is affected by changes in riparian plant communities, changes in soil organic matter, or variable stormwater flows and discharge. Furthermore, urban watersheds may behave differently than streams in less developed watersheds which have been more thoroughly studied (e.g. Peterson et al. 2001; Mulholland et al. 2004; Kaushal and Lewis 2005; Doheny and Baker 2018). Comparisons of streams in other regions and land use types will be critical in determining effectiveness of restoration and establishment of metrics to assess water quality improvements associated with stream and riparian restoration.

Performance among restoration designs and hydrologic conditions is likely to be highly variable, suggesting that stream restoration by itself is currently not adequate to mitigate for excess N inputs or to compensate for stream destruction and degradation (Craig et al. 2008) and that a comprehensive approach must be taken for watershed management (USEPA 2008) including reducing effective impervious surface coverage in uplands (Walsh et al. 2005) and repairing aging infrastructure (Doyle et al. 2008). A combination of restoration features and stormwater management (Reisinger et al. 2019) may improve long-term efficacy of restoration. Structural failures among restored stream features also underscores the importance of designing restoration projects to be stable at Q100, with a factor of safety (Hawley 2018).

Minebank Run is one of thousands of streams leading to the Chesapeake Bay representing about 5.2 km of stream length of the estimated 160,000 km of streams within the 166,000 km^2^ watershed of the Chesapeake Bay (USEPA 2009). Total cost of restoration at Minebank Run was $4.4 million (in 2005 US$; Baltimore County Department of Environmental Protection and Sustainability, unpublished). Employing this type of restoration solely for managing N is prohibitively expensive and likely will not address the impacts of future N loads and sources. While stream restoration is not the primary solution to N management in Chesapeake Bay, stream restoration has numerous cumulative, potential benefits that may justify the costs of such efforts, including, sediment and erosion control, protection of property, increased property values, fish and wildlife habitat and migration corridors, green space, stream temperature control, improved ecosystem metabolism, and maintenance of riparian zones.

Longevity or efficacy of restoration projects, especially under repeated storm effects or increasing urbanization, or from the effects of salinization and chemical cocktails (Cooper et al. 2014; Kaushal et al. 2020), is currently unknown. Storms after the restoration may have exceeded the channel design discharge for which the restoration at Minebank Run was engineered and/or the bankfull dimensions may have been too difficult to accurately identify (Sortman 2004). Soil instability and poor vegetation reestablishment also may lead to erosion during overbank flows (Sortman 2004). Natural channel design (NCD) restoration approaches integrate fluvial processes of “self-formed and self-maintained natural rivers” (Rosgen 2011). Because urban streams self-form in highly altered landscapes, applying NCD techniques in effort to reconstruct a stable reference reach yields unexpected outcomes and the geomorphologic evolution of such restored streams has not been fully documented. While NCD may produce a more functional stream in the sense of flashiness or nutrient uptake, metrics of success and failure have not yet been fully established and protocols for stream restoration intended to reduce N transport are evolving (Urban Stormwater Work Group 2020). Furthermore, ecosystem functions in urban streams such as nutrient uptake may be more resilient to disturbance, storm events, and floods because of functional redundancy of the microbial communities driving processes such as denitrification (Utz et al. 2016; Reisinger et al. 2017). Future studies should investigate effects of varying N loads, hydrologic residence times, hydrological connectivity, and seasonality on denitrification rates in restored streams and under different land uses and stream flow conditions (e.g. Tague et al. 2008). Further research on coupled restoration practices and stormwater management may be useful because it may be desirable to create conditions with high denitrification rates in urban areas where water from the landscape is concentrated (Pennino et al. 2016). In addition, the structure and function of urban streams can evolve over time with management or degradation (Kaushal et al. 2014a, b; Kaushal et al. 2015). To our knowledge, this study is among the first to look at long-term changes in the biogeochemistry and hydrology of a restored stream, and more work is needed to analyze long-term changes in material transport and retention in restored streams over time.

Restoration designs are heterogeneous efforts consisting of various components including bank re-shaping, bank stabilization, channel reconstruction, riparian re-vegetation, etc. Therefore, study designs that distinguish the influence of individual restoration components will help to identify those techniques that contribute most to nutrient uptake and other ecosystem functions of concern. Also, because in situ N uptake and transformation is notoriously difficult to measure (Groffman et al. 2006), studies that measure denitrification or surrogates of denitrification (e.g. oxidation–reduction potential, DO) at watershed scales and over time will be most useful in quantifying restoration effects. Finally, longterm monitoring will better elucidate short and long-term patterns of nutrient dynamics (Goodale et al. 2003, 2005, White et al. in prep). Therefore, long-term studies like ours (Pickett et al. 2001; Mayer et al. 2010a) are critical for understanding restoration effectiveness.

## Supplementary Material

1

## Figures and Tables

**Table 1:** Mixed-model ANOVA comparisons of nitrate (NO_3_^−^), dissolved organic carbon (DOC), and chloride (Cl^−^) concentrations (mg/L) in groundwater versus surface water at restored CVP and control Intervale (IV) sites during pre- and post-restoration time periods*. Water type (groundwater [GW] vs surface water [SW]) was used as a fixed factor while time (sampling period) was used as a random factor in the models. Table shows sample size (N), mean ($\bar{X}$), standard error (SE), degrees freedom (df), *F* statistics (F), and *p*-values (*p*). Statistical comparisons (*df, F, p*) represent a comparison of groundwater vs. surface water at each site during each time period.

				NO_3_^−^						DOC						Cl^−^					
treatment	time period	Site	GW/SW	N	mean	SE	*df*	*F*	*p*	N	mean	SE	*df*	*F*	*p*	N	mean	SE	*df*	*F*	*p*
restored	pre-restoration	CVP	GW	128	1.56	0.08	1,138	0.04	0.84	128	0.76	0.04	1,138	72.79	**<0.001**	128	119.5	5.88	1,138	3.27	0.07
			SW	15	1.61	0.03				15	1.78	0.16				15	150.2	14.15			
restored	post-restoration	CVP	GW	308*	1.33	0.06	1,340	2.63	0.11	314	0.79	0.03	1,346	149.54	**<0.001**	306	117.9	3.37	1,338	0.51	0.48
			SW	43	1.08	0.08				43	1.69	0.09				43	124.5	4.19			
control	pre-restoration	Intervale	GW	27	0.84	0.06	1,30	0.09	0.77	27	0.52	0.07	1,30	77.08	**<0.001**	26*	35.3	3.62	1,29	27.15	**<0.001**
			SW	8	0.88	0.10				8	1.95	0.21				8	77.2	15.15			
control	post-restoration	Intervale	GW	168	0.97	0.04	1,187	2.93	0.09	168	0.31	0.02	1,187	231.72	**<0.001**	168	31.2	0.36	1,187	271.66	**<0.001**
			SW	30	1.12	0.06				30	1.62	0.18				30	64.3	4.66			

* Six outliers (NO_3_^−^ > 6 mg/L) excluded from NO_3_^−^ analyses.

* One outlier (Cl^−^ > 400 mg/L) excluded from Cl^−^ analyses

**Table 2:** Mixed-model ANOVA comparisons of nitrate (NO_3_^−^), dissolved organic carbon (DOC), and chloride (Cl^−^) concentrations (mg/L) during pre- versus post-restoration periods at restored CVP and control Intervale (IV) sites in groundwater (GW) and surface water (SW). Site (CVP vs Intervale) was used as a fixed factor while time (sampling period) was used as a random factor in the models. Table shows sample size (N), mean ($\bar{X}$), standard error (SE), degrees freedom (df), *F* statistics (*F*), and *p*-values (*p*). Statistical comparisons (*df, F, p*) represent a comparison of pre- vs post-restoration at each site for each type of water.

				NO_3_^−^						DOC						Cl^−^					
treatment	time period	Site	GW/SW	N	mean	SE	*df*	*F*	*p*	N	mean	SE	*df*	*F*	*p*	N	mean	SE	*df*	*F*	*p*
restored	pre-restoration	CVP	GW	128	1.56	0.08	1,422	3.98	**0.05**	128	0.76	0.04	1,428	0.20	0.65	128	119.5	5.88	1,420	0.03	0.87
	post-restoration			308*	1.33	0.06				314	0.79	0.03				306	117.9	3.37			
restored	pre-restoration	CVP	SW	15	1.61	0.03	1,44	5.49	**0.02**	15	1.78	0.16	1,44	0.05	0.82	15	150.2	14.15	1,44	1.64	0.21
	post-restoration			43	1.08	0.08				43	1.69	0.09				43	124.5	4.19			
control	pre-restoration	Intervale	GW	27	0.84	0.06	1,181	1.84	0.18	27	0.52	0.07	1,181	10.71	**0.001**	26*	35.3	3.62	1, 180	1.48	0.26
	post-restoration			168	0.97	0.04				168	0.31	0.02				168	31.2	0.36			
control	pre-restoration	Intervale	SW	8	0.88	0.10	1,24	1.49	0.23	8	1.95	0.21	1,24	0.37	0.55	8	77.2	15.15	1,24	0.46	0.51
	post-restoration			30	1.12	0.06				30	1.62	0.18				30	64.3	4.66			

* Six outliers (NO_3_^−^ > 6 mg/L) excluded from NO_3_^−^ analyses.

* One outlier (Cl^−^ > 400 mg/L) excluded from Cl^−^ analyses

**Table 3:** Linear regression trends of nitrate (NO_3_^−^) concentrations (mg/L) and specific conductance (μS/cm) over time (2003–2008) in surface water collected by USGS during bi-weekly surveys at Minebank Run.

			NO_3_^−^ (mg/L)									Specific Conductance (uS/cm)								
treatment	Site	time period	N	mean	min	max	*R^2^*	coefficient	constant	*p* -value	trend	N	mean	min	max	*R^2^*	coefficient	constant	*p* -value	trend
restored	CVP	pre-restoration	76	1.59	0.23	4.43	0.42	0.0018	0.711	**<0.001**	**positive**	75	896.8	330	2140	0.05	0.278	763.4	**0.05**	**positive**
		construction	27	1.58	1.10	2.34	0.25	0.0018	−0.135	**0.008**	**positive**	26*	826.5	609	1027	0.23	0.647	221.2	**0.01**	**positive**
		post-restoration	170	1.44	0.57	2.48	0.08	−0.0003	1.939	**<0.001**	**negative**	170	945.2	235	2530	0.02	0.097	779.4	0.11	no trend
control	Intervale	construction	28	1.18	0.77	2.06	0.36	0.0022	−0.886	**0.001**	**positive**	28	540.3	353	1071	0.2	0.895	−302.03	**0.02**	**positive**
		post-restoration	171	1.11	0.53	2.19	0.05	−0.0002	1.428	**0.005**	**negative**	172	638.6	141	3540	0.004	0.07	5190.5	0.4	no trend

* One outlier (specific conductance > 5000 μS/cm) excluded from analyses

**Table 4:** ANOVA comparisons of nitrate (NO_3_^−^), dissolve organic carbon (DOC), and chloride (Cl^−^) concentrations (mg/L) in groundwater among stream features Minebank Run.

			NO_3_^−^			DOC			Cl^−^		
time period	feature	N	mean	SE	*post hoc* *	mean	SE	*post hoc* *	mean	SE	*post hoc* *
pre-restoration	citibank	22	2.60	0.30	a	0.55	0.12	a	59.8	6.3	a
	gravel bar	33	0.95	0.09	b	0.76	0.06	a	163.5	11.3	b
	stream channel	58	1.53	0.06	c	1.18	0.08	b	135.0	8.3	b,c
	terrace	24	1.51	0.08	c	0.61	0.05	a	111.4	10.3	c
post-restoration	citibank	3	0.91	0.09	a,b,c	0.94	0.07	a,b,c	74.3	8	a,b
	gravel bar	75	0.76	0.05	a	0.98	0.06	a	212.3	13.9	c
	stream channel	113	1.15	0.04	a,b	1.23	0.06	b	128.5	3.3	a
	terrace	55	1.48	0.08	b	0.60	0.05	c	99.1	5.2	a,b
	riprap	27	0.64	0.08	a	0.51	0.03	c	104.3	8.9	a,b
	oxbow	67	2.52	0.25	c	0.67	0.05	c	76.0	3.9	b

* Tukey’s post-hoc tests were performed to compare means during the pre-restoration and post-restoration periods, respectively. Means with the same letter are not significantly different *p* > 0.05

## References

[R1] Alexander RB, Smith RA, Schwarz GE (2000) Effect of stream channel size on the delivery of nitrogen to the Gulf of Mexico. Nature 403:758–761 10693802 10.1038/35001562

[R2] Altman SJ, Parizek RR (1995) Dilution of nonpoint-source nitrate in ground water. J Environmental Quality 24:707–718

[R3] APHA (1998) Standard Methods for the Examination of Water and Wastewater, 20th edn. American Public Health Association, Washington, DC

[R4] Baker DB, Richards RP, Loftus TT, Kramer JW (2004) A new flashiness index: Characteristics and applications to midwestern rivers and streams. J Am Water Resour Assoc 40:503–522

[R5] Beaulieu JJ, Golden HE, Knightes CD, Mayer PM, Kaushal SS, Pennino MJ, Arango CP, Balz DA, Elonen CM, Fritz KM, Hill BH (2015) Urban stream burial increases watershed scale nitrate export. PLoS One July 17, 2015; 10.1371/journal.pone.0132256 PMC4505844 26186731

[R6] Beaulieu JJ, Mayer PM, Kaushal SS, Pennino MJ, Arango CP, Balz DA, Canfield TJ, Elonen CM, Fritz KM, Hill BH, Ryu H, Santo Domingo JW (2014) Effects of urban stream burial on organic matter dynamics and reach scale nitrate retention. Biogeochemistry. 10.1007/s10533-014-9971-4

[R7] Bernhardt ES, Palmer MA, Allan JD, Alexander G, Barnas K, Brooks S, Carr J, Clayton S, Dahm C, Follstad-Shah J, Galat D, Gloss S, Goodwin P, Hart D, Hassett B, Jenkinson R, Katz S, Kondolf GM, Lake PS, Lave R, Meyer JL, O’Donnell TK, Pagano L, Powell B, Sudduth E (2005) Synthesizing U.S. river restoration efforts. Science 308:636–637 15860611 10.1126/science.1109769

[R8] Boulton AJ (2007) Hyporheic rehabilitation in rivers: restoring vertical connectivity. Freshw Biol 52:632–650

[R9] Brinson MM, Rheinhardt RD (1996) The role of reference wetlands in functional assessment and mitigation. Ecol Appl 6:69–76

[R10] Bukaveckas PA (2007) Effects of channel restoration on water velocity, transient storage, and nutrient uptake in a channelized stream. Environ Sci Technol 41:1570–1576 17396643 10.1021/es061618x

[R11] Cooper CA, Mayer PM, Faulkner BR (2014) Effects of road salts on groundwater and surface water dynamics of sodium and chloride in an urban restored stream. Biogeochemistry 121:149–166

[R12] Craig LS, Palmer MA, Richardson DC, Filoso S, Bernhardt ES, Bledsoe BP, Doyle MW, Groffman PM, Hassett BA, Kaushal SS, Mayer PM, Smith SM, Wilcock PR (2008) Stream restoration strategies for reducing river nitrogen loads. Front Ecol Environ 6:529–538

[R13] Doheny EJ, Baker SM (2018) Geomorphic characteristics of Tenmile Creek, Montgomery County, Maryland, 2014–16: U.S. Geological Survey Scientific Investigations Report 2018–5098, 34 p. 10.3133/sir20185098

[R14] Doheny EJ, Dillow JJA, Mayer PM, Striz EA (2012) Geomorphic responses to stream channel restoration at Minebank Run, Baltimore County, Maryland, 2002–08: U.S. Geological Survey Scientific Investigations Report 2012–5012, 61 p. http://pubs.usgs.gov/sir/2012/5012/

[R15] Doheny EJ, Starsoneck RJ, Mayer PM, Striz EA (2007) Pre-restoration geomorphic characteristics of Minebank Run, Baltimore County, Maryland, 2002–04. USGS Scientific Investigations Report 2007–5127

[R16] Doheny EJ, Starsoneck RJ, Striz EA, Mayer PM (2006) Watershed characteristics and pre-restoration surface-water hydrology of Minebank Run, Baltimore County, Maryland, water years 2002– 04: U.S. Geological Survey Scientific Investigations Report 2006–5179, 42 p

[R17] Doyle MW, Stanley EH, Havlick DG, Kaiser MJ, Steinbach G, Graf WL, Galloway GE, Riggsbee JA (2008) Aging Infrastructure and Ecosystem Restoration. Science 319:286–287 18202277 10.1126/science.1149852

[R18] Duan S, Mayer P, Kaushal S, Wessel B, Johnson T (2019) Regenerative stormwater conveyance (RSC) as a restoration approach to nutrient management may depend upon carbon quantity, quality, and source. Sci Total Environ 652:134–146 30359797 10.1016/j.scitotenv.2018.10.197 PMC6529187

[R19] Duerksen C, Snyder C (2005) Nature-Friendly Communities: Habitat Protection and Land Use Planning. Island Press, Washington, D.C., p 421

[R20] Elmore AJ, Kaushal SS (2008) Disappearing headwaters: Patterns of stream burial due to urbanization. Front Ecol Environ 6:308–312

[R21] Eshleman KN, Sabo RD, Kline KM (2013) Surface water quality is improving due to declining atmospheric N deposition. Environ Sci Technol. 10.1021/es4028748 24090248

[R22] Fennessy MS, Cronk JK (1997) The effectiveness and restoration potential of riparian ecotones for the management of nonpoint source pollution, particularly nitrate. Crit Rev Env Sci Tec 27:285–317

[R23] Filoso S, Palmer MA (2011) Assessing stream restoration effectiveness at reducing nitrogen export to downstream waters. Ecol Appl 21:1989–2006 21939039 10.1890/10-0854.1

[R24] Filoso S, Smith SMC, Williams MR, Palmer MA (2015) The efficacy of constructed stream−wetland complexes at reducing the flux of suspended solids to Chesapeake Bay. Environ Sci Technol 49:8986–8994. 10.1021/acs.est.5b00063 26181355 PMC9813913

[R25] Fink DF, Mitsch WJ (2007) Hydrology and nutrient biogeochemistry in a created river diversion oxbow wetland. Ecol Eng 30:93–102

[R26] Fisher H, Kloep F, Wilzceki S, Pusch MT (2005) A river’s liver – microbial processes within the hyporheic zone of a large lowland river. Biogeochemistry 76:349–371

[R27] Gift DM, Groffman PM, Kaushal SS, Mayer PM (2010) Root biomass, organic matter and denitrification potential in degraded and restored urban riparian zones. Restor Ecol 18:113–120

[R28] Goodale LC, Aber JD, Vitousek PM (2003) An unexpected nitrate decline in New Hampshire streams. Ecosystems 6:75–86

[R29] Goodale LC, Aber JD, Vitousek PM, McDowell WH (2005) Longterm decreases in stream nitrate: successional causes unlikely; possible links to DOC? Ecosystems 8:334–337

[R30] Grant SB, Azizian M, Cook P, Boano F, Rippy MA (2018) Factoring stream turbulence into global assessments of nitrogen pollution. Science 359:1266–1269 29590077 10.1126/science.aap8074

[R31] Grimm NB, Sheibley RW, Crenshaw CL, Dahm CN, Roach WJ, Zeglin LH (2005) N retention and transformation in urban streams. J N Am Benthol Soc 24:626–642

[R32] Groffman PM, Altabet MA, Böhlke JK, Butterbach-Bahl K, David MB, Firestone MK, Giblin AE, Kana TM, Nielsen LP, Voytek MA (2006) Methods for measuring denitrification: Diverse approaches to a difficult problem. Ecol Appl 16:2091–2122 17205891 10.1890/1051-0761(2006)016[2091:mfmdda]2.0.co;2

[R33] Groffman PM, Bain DJ, Band LE, Belt KT, Brush GS, Grove JM, Pouyat RV, Yesilonis IC, Zipperer WC (2003) Down by the riverside: urban riparian ecology. Front Ecol Environ 6:315–321

[R34] Groffman PM, Boulware NJ, Zipperer WC, Pouyat RV, Band LE, Colosimo MF (2002) Soil nitrogen cycling processes in urban riparian zones. Environ Sci Technol 36:4547–4552 12433163 10.1021/es020649z

[R35] Groffman PM, Crawford MK (2003) Denitrification potential in urban riparian zones. J Environmental Quality 32:1144–1149 10.2134/jeq2003.1144 12809317

[R36] Groffman PM, Dorsey AM, Mayer PM (2005) N processing within geomorphic structures in urban streams. J North American Benthological Society 24:613–625

[R37] Gücker B, Boechat IG (2004) Stream morphology controls ammonium retention in tropical headwaters. Ecology 85:2818–2827

[R38] Hammersmark CT, Rains MC, Mount JF (2008) Quantifying the hydrological effects of stream restoration in a montane meadow, northern California, USA. River Res Appl 24:735–753

[R39] Harrison MD, Groffman PM, Mayer PM, Kaushal SS, Newcomer TA (2011) Denitrification in alluvial wetlands in an urban landscape. J Environ Qual 40:634–646 21520770 10.2134/jeq2010.0335

[R40] Harrison MD, Groffman PM, Mayer PM, Kaushal SS (2012a) Nitrate removal in two relict oxbow urban wetlands: A 15N mass-balance approach. Biogeochemistry 111:647–660

[R41] Harrison MD, Groffman PM, Mayer PM, Kaushal SS (2012) Microbial biomass and activity in geomorphic features in forested and urban restored and degraded streams. Ecol Eng 38:1–10

[R42] Harrison MD, Miller AJ, Groffman PM, Mayer PM, Kaushal SS (2014) Hydrologic controls on nitrogen and phosphorous dynamics in relict wetlands adjacent to an urban restored stream. J Am Water Resour Assoc 50:1365–1382

[R43] Hassett B, Palmer M, Bernhardt E, Smith S, Carr J, Hart D (2005) Restoring watersheds project by project: Trends in Chesapeake Bay tributary restoration. Front Ecol Environ 3:259–267

[R44] Hawley RJ (2018) Making stream restoration more sustainable: A geomorphically, ecologically, and socioeconomically principled approach to bridge the practice with the science. Bioscience 68:517–528 30002563 10.1093/biosci/biy048 PMC6037085

[R45] Hedin LO, von Fischer JC, Ostrom NE, Kennedy BP, Brown MG, Robertson GP (1998) Thermodynamic constraints on nitrogen transformations and other biogeochemical processes at soilstream interfaces. Ecology 79:684–703

[R46] Hill AR, Labadia CF, Sanmugadask K (1998) Hyporheic zone hydrology and nitrogen dynamics in relation to the streambed topography of a N rich stream. Biogeochemistry 42:285–310

[R47] Hirsch RM, De Cicco LA (2015) User Guide to Exploration and Graphics for RivEr Trends (EGRET) and data Retrieval: R Packages for Hydrologic Data, USGS. Chapter 10 of Section A, Statistical Analysis, Book 4, Hydrologic Analysis and Interpretation, Techniques and Methods 4–A10, Version 2.0, February 2015, U.S

[R48] Hirsch RM, Moyer DL, Archfield SA (2010) Weighted regressions on time, discharge, and season (WRTDS), with an application to Chesapeake Bay river inputs 1. J Am Water Resour Assoc 46:857–880 22457569 10.1111/j.1752-1688.2010.00482.x PMC3307614

[R49] Hudson PF, Heitmuller FT, Leitch MB (2012) Hydrologic connectivity of oxbow lakes along the lower Guadalupe River, Texas: The influence of geomorphic and climatic controls on the “flood pulse concept.” J Hydrol 414:174–183

[R50] Jacobson RB, Lindner G, Bitner C (2015) The role of floodplain restoration in mitigating flood risk, Lower Missouri River, USA. Pages 203–243 in “Geomorphic approaches to integrated floodplain management of lowland fluvial systems in North America and Europe”, Hudson Paul F. and Middlekoop Hans eds. Springer

[R51] Jones JB Jr, Holmes RM (1996) Surface-subsurface interaction in stream ecosystems. Trends Ecol Evol 11:239–242 21237825 10.1016/0169-5347(96)10013-6

[R52] Kasahara T, Hill AR (2006) Hyporheic exchange flows induced by constructed riffles and steps in lowland streams in southern Ontario, Canada. Hydrol Process 20:4287–4305

[R53] Kaushal SS, Belt KT (2012) The urban watershed continuum: evolving spatial and temporal dimensions. Urban Ecosystems 15:409–435

[R54] Kaushal SS, Delaney-Newcomb K, Findlay SEG, Newcomer TA, Duan S, Pennino MJ, Sivirichi GM, Sides-Raley AM, Walbridge MR, Belt KT (2014a) Longitudinal patterns in carbon and nitrogen fluxes and stream metabolism along an urban watershed continuum. Biogeochemistry 121:23–44

[R55] Kaushal SS, Groffman PM, Mayer PM, Striz EA, Doheny EJ, Gold AJ (2008) Effects of stream restoration on denitrification at the riparian-stream interface of an urbanizing watershed of the mid-Atlantic U.S. Ecol Appl 18:789–804 18488635 10.1890/07-1159.1

[R56] Kaushal SS, Lewis WM Jr (2005) Transport and fate of dissolved organic nitrogen in minimally disturbed streams. Biogeochemistry 74:303–321

[R57] Kaushal SS, McDowell WH, Wollheim WM (2014b) Tracking evolution of urban biogeochemical cycles: past, present, and future. Biogeochemistry 121:1–21

[R58] Kaushal SS, McDowell WH, Wollheim WM, Newcomer Johnson TA, Mayer PM, Belt KT, Pennino MJ (2015) Urban Evolution: The Role of Water. Water 7:4063–4087

[R59] Kaushal SS, Wood KL, Galella JG, Giona AM, Haq S, Goodling PJ, Haviland KA, Reimer JE, Morel CJ, Wessel B, Nguyen W, Hollingsworth JW, Mei K, Leal J, Widmer J, Sharif R, Mayer PM, Newcomer Johnson TA, Newcomb KD, Smith E, Belt KT (2020) Making urban ‘chemical cocktails’ – Evolving geochemical processes across the Periodic Table of elements. Appl Geochem. 10.1016/j.apgeochem.2020.104632 PMC7970522 33746355

[R60] Kaye JP, Groffman PM, Grimm NB, Baker LA, Pouyat RV (2006) A distinct urban biogeochemistry? Trends Ecol Evol 21:192–199 16701085 10.1016/j.tree.2005.12.006

[R61] Kemp MJ, Dodds WK (2002) The influence of ammonium, nitrate, and dissolved oxygen concentrations on uptake, nitrification, and denitrification rates associated with prairie stream substrata. Limnol Oceanogr 47:1380–1393

[R62] Klocker CA, Kaushal SS, Groffman PM, Mayer PM, Morgan RP (2009) Nitrogen uptake and denitrification in a restored urban stream. Aquat Sci 71:411–424

[R63] Konrad CP, Booth DB, Burges SJ (2005) Effects of urban development in the Puget Lowland, Washington, on interannual streamflow patterns: Consequences for channel form and streambed disturbance. Water Resour Res 41:W07009. 10.1029/2005WR004097

[R64] Lazar JG, Gold AJ, Addy K, Mayer PM, Forshay KJ, Groffman PM (2014) Instream large wood: Denitrification hotspots with low N2O production. J Am Water Resour Assoc 50:615–625

[R65] Linker LC, Dennis R, Shenk GW, Batiuk RA, Grimm J, Wang P (2013) Computing atmospheric nutrient loads to the Chesapeake Bay watershed and tidal waters. J Am Water Resour Assoc 1–17. 10.1111/jawr.12112

[R66] Loperfido JV, Noe GB, Jarnagin ST, Hogan DM (2014) Effects of distributed and centralized stormwater best management practices and land cover on urban stream hydrology at the catchment scale. J Hydrol 519:2584–2595

[R67] Lovett GM, Trayno MM, Pouyat RV, Carreiro MM, Zhu W-X, Baxter JW (2000) Atmospheric deposition to oak forests along an urbanrural gradient. Environ Sci Technol 34:4294–4300

[R68] Mayer P, Grimm N, Lepczyk C, Pickett S, Pouyat R, Warren P (2010a) Urban ecosystems research joins mainstream ecology. Nature 467:153. 10.1038/467153b 20829773

[R69] Mayer PM, Groffman PM, Striz E, Kaushal SS (2010b) Nitrogen dynamics at the ground water-surface water interface of a degraded urban stream. J Environ Qual 39:810–823 20400577 10.2134/jeq2009.0012

[R70] Mayer PM, Reynolds SK, McCutchen MD, Canfield TJ (2007) Metaanalysis of nitrogen removal in riparian buffers. J Environ Qual 36:1172–1180 17596626 10.2134/jeq2006.0462

[R71] Mayer PM, Schechter SP, Kaushal SS, Groffman PM (2013) Effects of stream restoration on nitrogen removal and transformation in urban watersheds: Lessons from Minebank Run, Baltimore, Maryland. Watershed Science Bulletin (Spring) Vol. 4, Issue 1, online: https://www.cwp.org/watershed-science-bulletin-past-issues/

[R72] Mayer PM, Striz E, Shedlock R, Doheny Groffman P (2003) The effects of ecosystem restoration on nitrogen processing in an urban mid-Atlantic piedmont stream. Pp. 536–541 in Renard, Kenneth G, McElroy, Stephen A, Gburek, William J, Canfield, H. Evan and Scott, Russell L, eds. First Interagency Conference on Research in the Watersheds, October 27–30, 2003. U.S. Department of Agriculture, Agricultural Research Service

[R73] McClain ME, Boyer EW, Dent CL, Gergel SE, Grimm NB, Groffman PM, Hart SC, Harvey JW, Johnston CA, Mayorga E, McDowell WH, Pinay G (2003) Biogeochemical hot spots and hot moments at the interface of terrestrial and aquatic ecosystems. Ecosystems 6:301–312

[R74] Medalie L, Hirsch RM, Archfield SA (2012) Use of flow-normalization to evaluate nutrient concentration and flux changes in Lake Champlain tributaries, 1990–2009. J Great Lakes Res 38:58–67

[R75] Meierdiercks KL, Smith JA, Baeck ML, Miller AJ (2010) Analyses of urban drainage network structure and its impact on hydrologic response. J Am Water Resour Assoc 46:932–943

[R76] Mulholland PJ, Helton AM, Poole GC, Hall RO Jr, Hamilton SK, Peterson BJ, Tank JL, Ashkenas LR, Cooper LW, Dahm CN, Dodds WK, Findlay SEG, Gregory SV, Grimm NB, Johnson SL, McDowell WH, Meyer JL, Valett HM, Webster JR, Arango CP, Beaulieu JJ, Bernot MJ, Burgin AJ, Crenshaw CL, Johnson LT, Niederlehner BR, O’Brien JM, Potter JD, Sheibley RW, Sobota DJ, Thomas SM (2008) Stream denitrification across biomes and its response to anthropogenic nitrate loading. Nature 452:202–206 18337819 10.1038/nature06686

[R77] Mulholland PJ, Valett HM, Webster JR, Thomas SA, Cooper LW, Hamilton S, Peterson BJ (2004) Stream denitrification and total nitrate uptake lengths measured using 15N tracer addition approach. Limnol Oceanogr 49:809–820

[R78] Munn NL, Meyer JL (1990) Habitat-specific solute retention in two small streams: an intersite comparison. Ecology 71:2069–2082

[R79] Newcomer TA, Kaushal SS, Mayer PM, Shields AR, Canuel EA, Groffman PM, Gold AJ (2012) Influence of natural & novel organic carbon sources on denitrification in forested, degraded-urban, & restored streams. Ecol Monogr 82:449–466

[R80] Newcomer-Johnson T, Kaushal SS, Mayer PM, Smith R, Sivirichi G (2016) Nutrient Retention in Restored Streams and Rivers: A Global Review and Synthesis. Water 8 (4), 116. 10.3390/w8040116

[R81] Palmer M (2009) Reforming watershed restoration: Science in need of application and applications in need of science. Estuaries & Coasts. 10.1007/s12237-008-9129-5

[R82] Palmer MA, Filoso S, Fanelli RM (2014) From ecosystems to ecosystem services: Stream restoration as ecological engineering. Ecol Eng 65:62–70

[R83] Paul MJ, Meyer JL (2001) Streams in the urban landscape. Annu Rev Ecol Syst 32:333–365

[R84] Pennino MJ, Kaushal SS, Beaulieu JJ, Mayer PM, Arango CP (2014) Effects of urban stream burial on nitrogen uptake and ecosystem metabolism: implications for watershed nitrogen and carbon fluxes. Biogeochemistry 121:247–269

[R85] Pennino MJ, Kaushal SS, Mayer PM, Utz RM, Cooper CA (2016) Stream restoration and sewers impact sources and fluxes of water, carbon, and nutrients in urban watersheds. Hydrol Earth Syst Sci 20:3419–3439

[R86] Peterson BJ, Wollheim WM, Mulholland PJ, Webster JR, Meyer JL, Tank JL, Marti E, Bowden WB, Valett HM, Hershey AE, McDowell WH, Dodds WK, Hamilton SK, Gregory S, Morrall DD (2001) Control of nitrogen export from watersheds by headwater streams. Science 292:86–90 11292868 10.1126/science.1056874

[R87] Pickett STA, Cadenasso ML, Grove JM, Nilon CH, Pouyat RV, Zipperer WC, Costanza R (2001) Urban ecological systems: linking terrestrial ecological, physical, and socioeconomic components of metropolitan areas. Annu Rev Ecol Syst 32:127–157

[R88] Poff NL, Bledsoe BP, Cuhaciyan CO (2006) Hydrologic variation with land use across the contiguous United States: Geomorphic and ecological consequences for stream ecosystems. Geomorphology 79:264–285

[R89] Puls RW, Barcelona MJ (1996) Ground Water Issue: Low-Flow (Minimal Drawdown) Ground-Water Sampling Procedures. USEPA, National Risk Management Research Lab, Ada, OK, USA. PB-97–118822/XAB; EPA-540/S-95/504

[R90] Reisinger AJ, Rosi EJ, Bechtold HA, Doody TR, Kaushal SS, Groffman PM (2017) Recovery and resilience of urban stream metabolism following Superstorm Sandy and other floods. Ecosphere 8(4):e01776. 10.1002/ecs2.1776

[R91] Reisinger AJ, Woytowitz E, Majcher E, Rosi EJ, Belt KT, Duncan JM, Kaushal SS, Groffman PM (2019) Changes in long-term water quality of Baltimore streams are associated with both gray and green infrastructure. Limnol Oceanogr 64:S60–S76. 10.1002/lno.10947

[R92] Roberts BJ, Mulholland PJ, Houser JN (2007) Effects of upland disturbance and instream restoration on hydrodynamics and ammonium uptake in headwater streams. J North American Benthological Society 24:613–625

[R93] Rosgen D (1996) Applied River Morphology. Wildland Hydrology, Pagosa Springs, CO

[R94] Rosgen DL (2011) Natural Channel Design: Fundamental Concepts, Assumptions, and Methods. In: Simon A, Bennett SJ, Castro JM (eds) Stream Restoration in Dynamic Fluvial Systems: Scientific Approaches, Analyses, and Tools, Geophysical Monograph Series 194. American Geophysical Union, Washington, D.C., pp 69–93

[R95] Sholtes JS, Doyle MW (2011) Effect of channel restoration on flood wave attenuation. J Hydraul Eng ASCE 137:196–208

[R96] Smith BK, Smith JA, Baeck M, Villarini LG, Wright DB (2013) Spectrum of storm event hydrologic response in urban watersheds. Water Resour Res 49:2649–2663

[R97] Sobczak WV, Findlay S (2002) Variation in bioavailability of dissolved organic carbon among stream hyporheic flowpaths. Ecology 83:3194–3209

[R98] Sobczak WV, Findlay S, Dye S (2002) Relationships between DOC bioavailability and nitrate removal in an upland stream: An experimental approach. Biogeochemistry 62:309–327

[R99] Sortman VL (2004) Complications with Urban Stream Restorations Mine Bank Run: A Case Study. ACSE Publications. 10.1061/40695(2004)19

[R100] Sudduth EB, Hassett BA, Cada P, Bernhardt ES (2011) Testing the Field of Dreams Hypothesis: functional responses to urbanization and restoration in stream ecosystems. Ecol Appl 21:1972–1988 21939038 10.1890/10-0653.1

[R101] Stanley EH, Doyle MW (2002) A geomorphic perspective on nutrient retention following dam removal. Bioscience 52:693–701

[R102] Striz EA, Mayer PM (2008) Assessment of near-stream ground watersurface water interaction (GSI) of a degraded stream before restoration. EPA 600/R-07/058, U.S. Environmental Protection Agency, Washington, DC

[R103] Tague C, Valentine S, Kotchen M (2008) Effect of geomorphic channel restoration on streamflow and groundwater in a snowmelt-dominated watershed. Water Resources Research 44: Article Number: W10415. 10.1029/2007WR006418

[R104] Taylor PG, Townsend AR (2010) Stoichiometric control of organic carbon–nitrate relationships from soils to the sea. Nature 464:1178–1181 20414306 10.1038/nature08985

[R105] Thompson J, Pelc CE, Brogan WR III, Jordan TE (2018) The multiscale effects of stream restoration on water quality. Ecol Eng 124:7–18

[R106] Underwood AJ (1992) Beyond BACI: the detection of environmental impacts on populations in the real, but variable, world. J Exp Mar Biol Ecol 161:145–178

[R107] Urban Stormwater Work Group: Altland D, Becraft C, Berg J, Burch J, Clearwater D, Crawford S, Doll B, Geratz J, Hanson J, Hartranft J, Hottenstein J, Kaushal S, Lowe S, Mayer P, Noe G, Scott D, Stack B (2020) Consensus Recommendations for Improving Protocols 2 and 3 on Effect of Stream and Floodplain Restoration Projects Built for Pollutant Removal Credit. Chesapeake Bay Program. 93 pp. https://www.chesapeakebay.net/documents/FINAL_Approved_Group_4_Memo_10.27.20.pdf

[R108] USEPA (1983) Methods for chemical analysis of water and wastes. EPA 841-B-08–002. Washington, DC

[R109] USEPA (2006) Baltimore County Stream Restoration Improves Quality of Life. EPA/903/F-06/008. Ft. Meade, MD

[R110] USEPA (2008) Handbook for Developing Watershed Plans to Restore and Protect Our Waters. EPA-600/4–79–020. Cincinnati OH

[R111] USEPA (2009) Bay Barometer: A health and restoration assessment of the Chesapeake Bay and Watershed in 2008. EPA-903-R-09–001, Chesapeake Bay Program

[R112] Utz RM, Hopkins KG, Beesley L, Booth DB, Hawley RJ, Baker ME, Freeman MC, Jones KL (2016) Ecological resistance in urban streams: the role of natural and legacy attributes. Freshwater Science 35:380–397

[R113] Vidon P, Allan C, Burns D, Duval TP, Gurwick N, Inamdar S, Lowrance R, Okay J, Scott D, Sebestyen S (2010) Hot spots and hot moments in riparian zones: Potential for improved water quality management. J Am Water Resour Assoc 1–21. 10.1111/j.1752-1688.2010.00420.x

[R114] Violin CR, Cada P, Sudduth EB, Hassett BA, Penrose DL, Bernhardt ES (2011) Effects of urbanization and urban stream restoration on the physical and biological structure of stream ecosystems. Ecol Appl 21:1932–1949 21939035 10.1890/10-1551.1

[R115] Walsh CJ, Roy AH, Feminella JW, Cottingham PD, Groffman PM, Morgan RP II (2005) The urban stream syndrome: current knowledge and the search for a cure. J North American Benthological Society 24:706–723

[R116] White DP, Newcomer-Johnson TN (in prep) A review of habitat restoration projects across Great Lakes Areas of Concern: Variability in actions, goals, monitoring, and outcomes

[R117] Wollheim WM, Pellerin BA, Vörösmarty CJ, Hopkinson CS (2005) N retention in urbanizing headwater catchments. Ecosystems 8:871–884

[R118] Woltemade CJ, Potter KW (1994) A watershed modeling analysis of fluvial geomorphologic influences on flood peak attenuation. Water Resour Res 30:1933–1942

[R119] Wood KL, Kaushal SS, Vidon PG, Mayer PM, Galella JG (This issue) Tree trade-offs in stream restoration projects: impact on riparian groundwater quality10.1007/s11252-021-01182-8 PMC9611097 36310660

