# Supplementary material for: Long-term assessment of floodplain reconnection as a stream restoration approach for managing nitrogen in ground and surface waters
Source: Urban Ecosyst. Author manuscript; Available in PMC 2023 Jun 1. (PMC8935950; doi:10.1007/s11252-021-01199-z)

Supplemental Information

Figure S1. Drought and discharge affected NO_3^- (a) and TN (b) concentrations

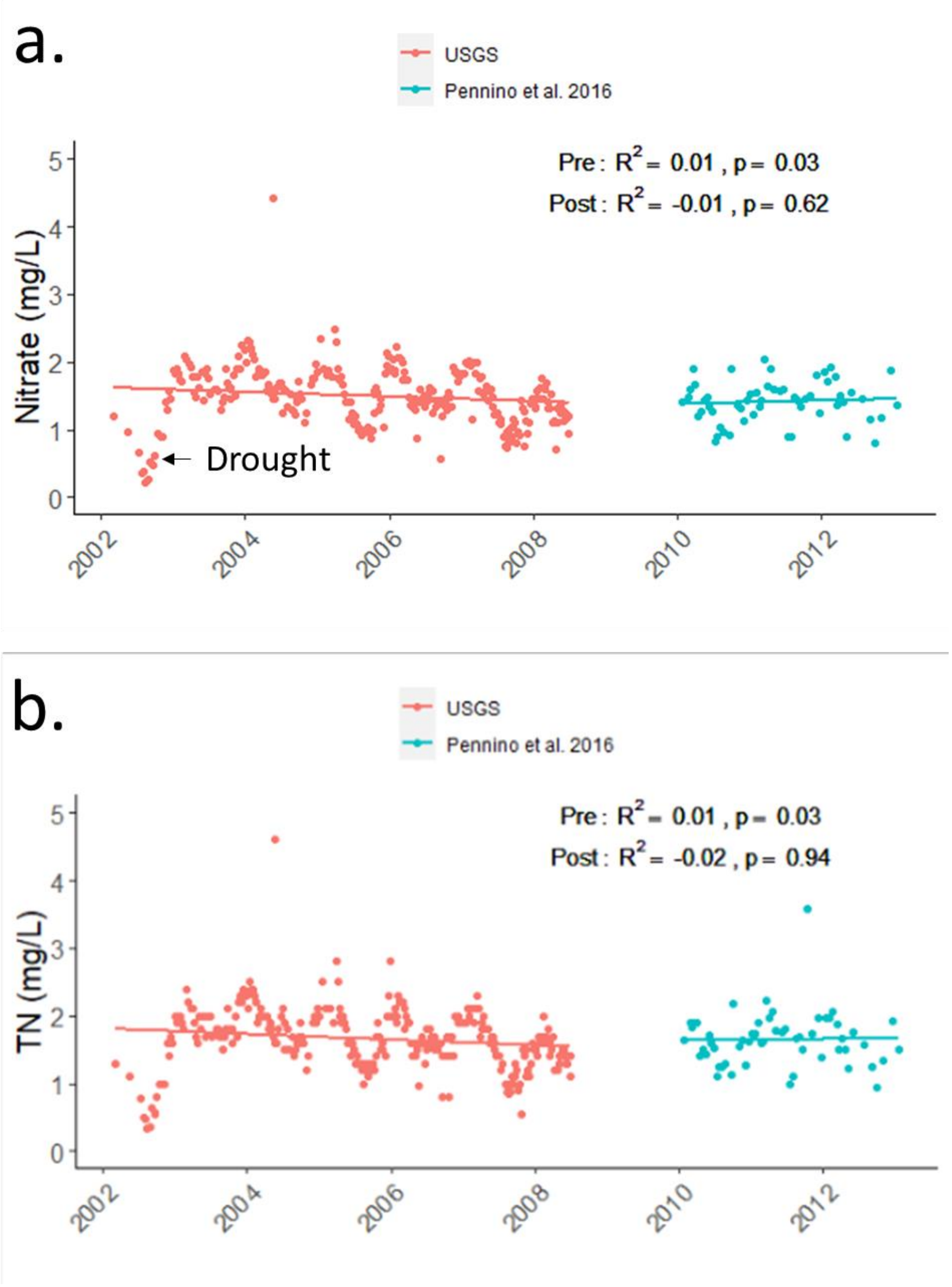

Figure S2. The average monthly Precipitation Flashiness Index: a) from 2002-2008 and b) from 2002-2012.

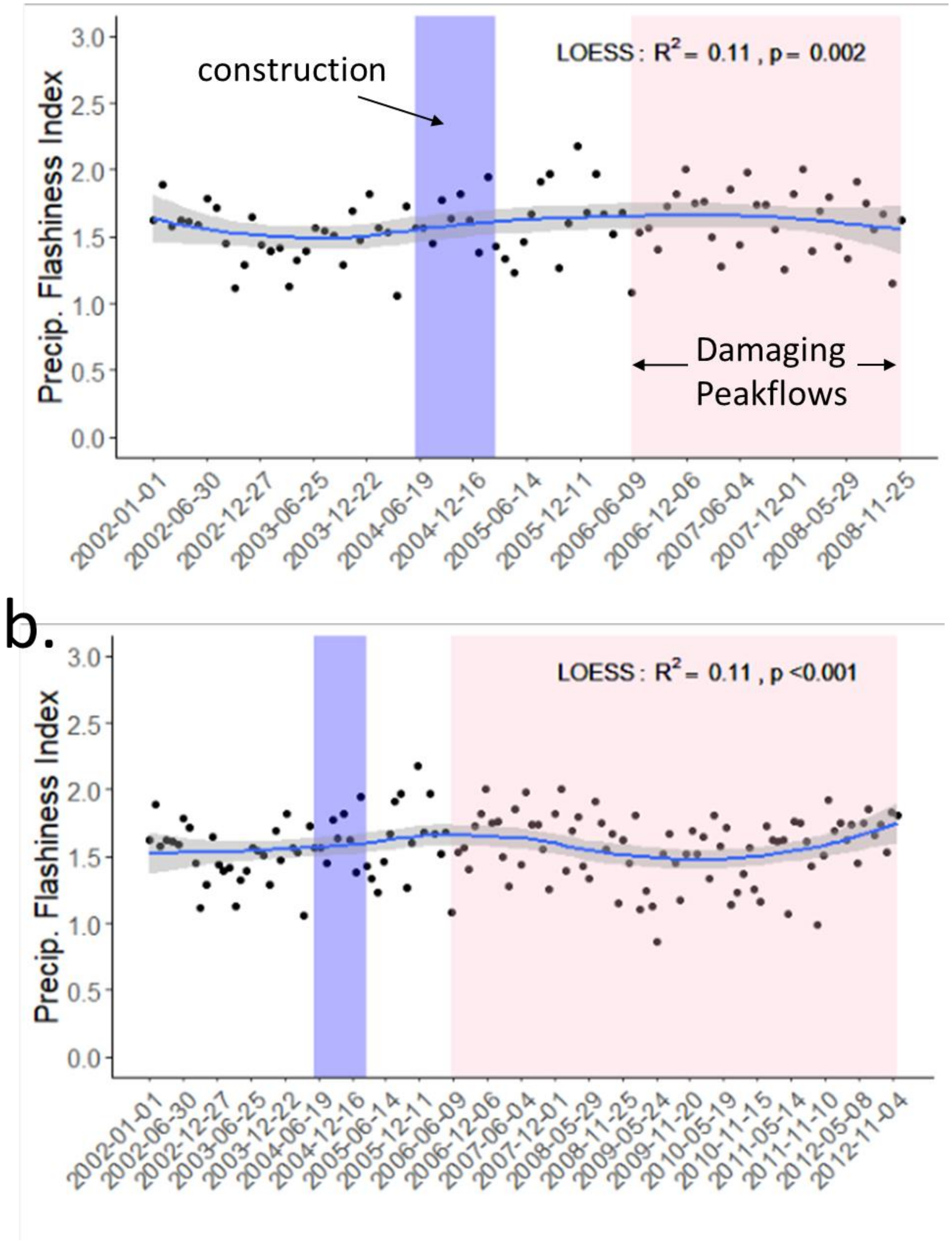

Figure S3. Precipitation Flashiness compared during pre- and post-restoration periods for (a) 2002-2008 and (b) 2002-2012.

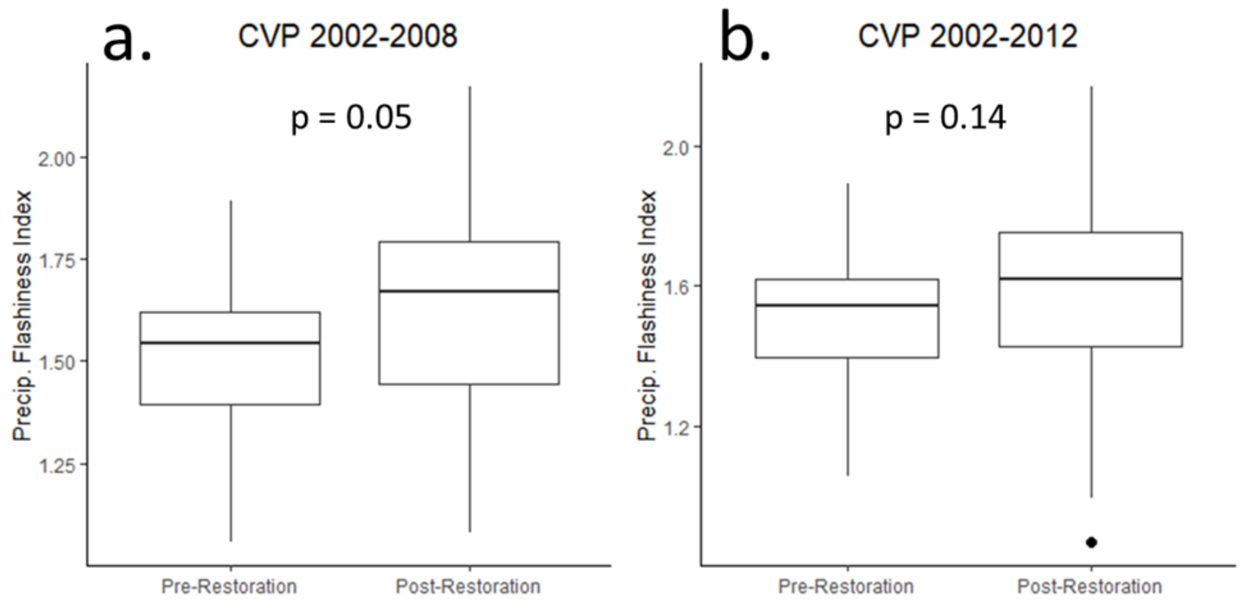

Figure S4a and b. Stream channel evolution after restoration. (a) April 2006 is 14 months after the restoration. May 2014 is 9 years, 3 months after restoration. Note bank erosion and formation of gravel bar. Before and after photos were taken from approximately the same location. A tree, road, and house are identified in each photo for orientation. (b) February 2021 is 16 years after restoration. Note bank erosion, exposed roots, and movement of cobble. Before and after photos were taken from approximately the same location. A barn and shed are identified in each photo for orientation. Note bank erosion has worsened. (Feb 2021 photo from R. Shedlock)

a

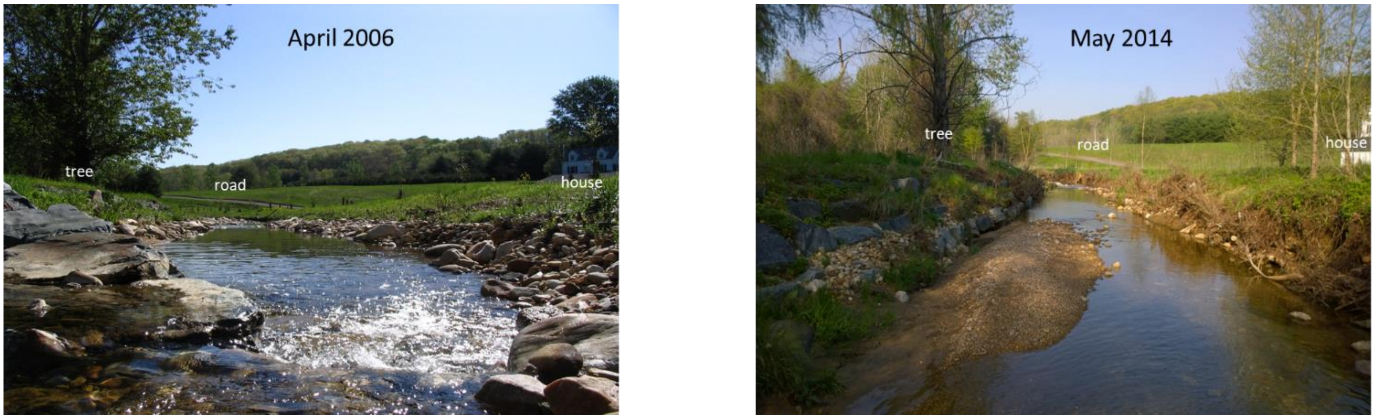

b

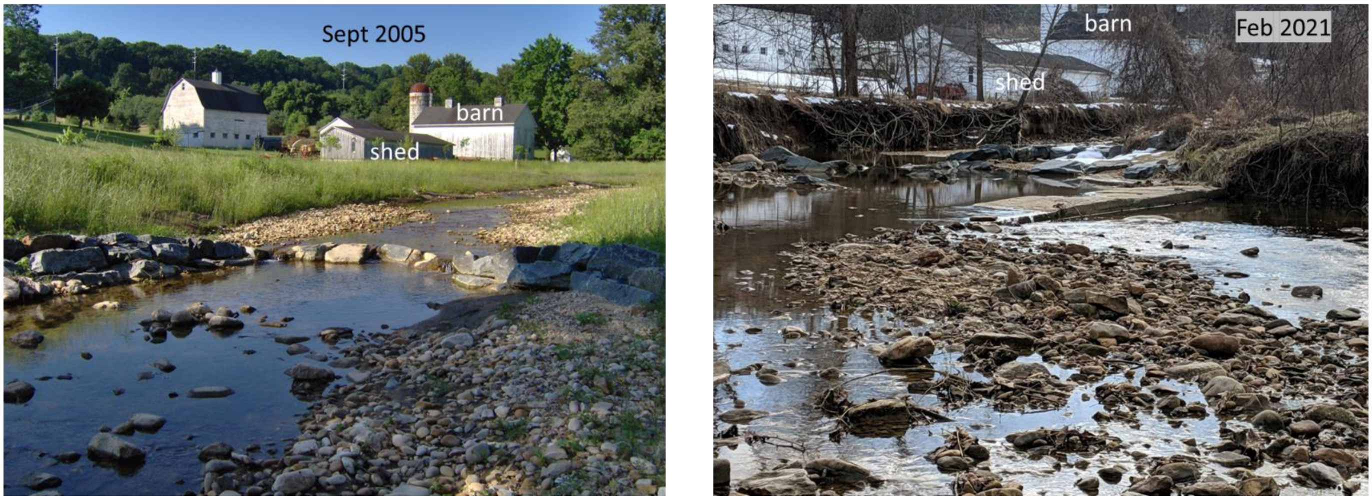

Supplement: 1 [file NIHMS1771514-supplement-1.pdf]
